# CCTα and CCTδ Chaperonin Subunits Are Essential and Required for Cilia Assembly and Maintenance in *Tetrahymena*

**DOI:** 10.1371/journal.pone.0010704

**Published:** 2010-05-18

**Authors:** Cecilia Seixas, Teresa Cruto, Alexandra Tavares, Jacek Gaertig, Helena Soares

**Affiliations:** 1 Instituto Gulbenkian de Ciência, Oeiras, Portugal; 2 Department of Cellular Biology, University of Georgia, Athens, Georgia, United States of America; 3 Escola Superior de Tecnologia da Saúde de Lisboa, Lisboa, Portugal; Baylor College of Medicine, United States of America

## Abstract

**Background:**

The eukaryotic cytosolic chaperonin CCT is a hetero-oligomeric complex formed by two rings connected back-to-back, each composed of eight distinct subunits (CCTα to CCTζ). CCT complex mediates the folding, of a wide range of newly synthesised proteins including tubulin (α, β and γ) and actin, as quantitatively major substrates.

**Methodology/Principal Findings:**

We disrupted the genes encoding CCTα and CCTδ subunits in the ciliate *Tetrahymena*. Cells lacking the zygotic expression of either CCTα or CCTδ showed a loss of cell body microtubules, failed to assemble new cilia and died within 2 cell cycles. We also show that loss of CCT subunit activity leads to axoneme shortening and splaying of tips of axonemal microtubules. An epitope-tagged CCTα rescued the gene knockout phenotype and localized primarily to the tips of cilia. A mutation in CCTα, G346E, at a residue also present in the related protein implicated in the Bardet Biedel Syndrome, BBS6, also caused defects in cilia and impaired CCTα localization in cilia.

**Conclusions/Significance:**

Our results demonstrate that the CCT subunits are essential and required for ciliary assembly and maintenance of axoneme structure, especially at the tips of cilia.

## Introduction

Cilia are conserved organelles with important sensory and motile functions. Defects in cilia have been associated with a large number of human diseases, collectively known as ciliopathies. Cilia have a microtubule-based axoneme that is anchored to the basal body. The axoneme is typically composed of 9 doublet-microtubules arranged as a peripheral ring. Motile cilia usually have a pair of singlet microtubules at the center of the axoneme. The assembly and maintenance of cilia is dependent on bidirectional trafficking of protein complexes between the cell basal body and the cilia tip, the activity known as intraflagellar transport (IFT) [1]. Kinesin-2 motors move IFT cargo from the cell body to the tip of cilia, while recycled components are returned to the basal body by cytoplasmic dynein 1b motors [2], [3], [4], [5].

The presence of different classes of molecular chaperones has been reported in cilia of diverse organisms. In *Chlamydomonas*, Hsp40 and Hsp70 were found in flagella [6], [7], Hsp40 and the CCTα/TCP-1 subunit of the cytosolic chaperonin CCT were found in cilia of sea urchin embryos [8], [9] and Hsp70 and Hsp90 were detected in cilia of *Tetrahymena* [10]. These chaperones could have a role in ensuring that the ciliary proteins preserve their native functional conformation during and after ciliogenesis, possibly by participating in the assembly or maintenance of large ciliary protein complexes. In fact, Hsp40 is a component of the radial spoke complex in sperm flagella of the ascidian *Ciona intestinalis* [11] and flagella of *Chlamydomonas*, where it may be involved in interactions between the radial spoke and central microtubules [12]. A mutation in BBS6, a protein related to CCTα, causes the Bardel Biedl Syndrome, a disease associated with defects in the function of cilia [13].

We have reported that in *Tetrahymena*, the expression of CCT chaperonin subunit genes is up-regulated during cilia regeneration following deciliation [14], [15] and CCTα, δ, ε and η subunits localize to growing and full-length cilia of *Tetrahymen*a [16]. CCT is a hetero-oligomeric complex formed by two rings connected back-to-back, each composed of eight distinct subunits (CCTα to CCTζ) [17]. Each CCT subunit consists of three domains: an equatorial domain containing an ATP-binding site, an apical domain that interacts with the target protein, and the intermediate domain that connects the apical and equatorial domain. The apical domain contains a helical protrusion [18], which is involved in opening and closing the central cavity of the chaperonin. The full size CCT complex mediates the folding, driven by ATP binding and hydrolysis, of a wide range of newly synthesised proteins including tubulin (α, β and γ) [19], [20], [21] and actin [22], [23] as quantitatively major substrates.

In this study, we investigate the role of CCTα and CCTδ subunits in *Tetrahymena*. We show that both CCTα and CCTδ subunits are required for survival of *Tetrahymena*. Cells lacking expression of CCT subunits, before their death, show dramatic alterations in the microtubule cytoskeleton and cilia. An epitope-tagged CCTα rescued the gene knockout phenotype and revealed that CCTα is a ciliary protein that is important for the maintenance of cilia tip integrity. We also show that a mutation of a conserved amino acid in CCTα that is also present in BBS6, a cilia-specific CCTα-related protein, affects the cytoskeleton and cilia. Collectively, our data show that CCT components are essential in a ciliated cell type, and that the referred CCT subunits play specific roles in ciliary assembly and maintenance.

## Results

### CCTα and CCTδ are essential in *Tetrahymena*

**Figure 1:**
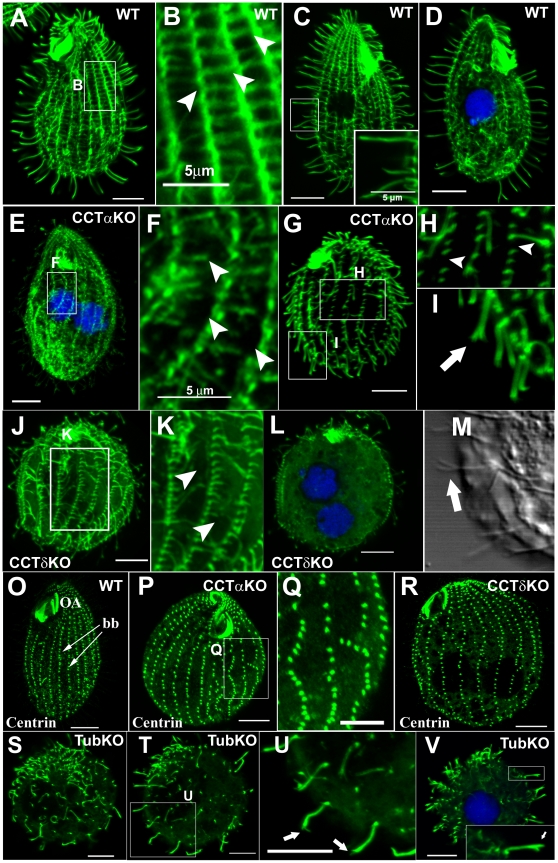
CCT subunits are required for assembly of axonemal, cortical and cell body microtubules. Confocal immunofluorescence of α-tubulin (**A** to **L** and **S** to **V**) and centrin (**O** to **R**) in wildtype, CCTα-KO, CCTδ-KO and tubulin-KO *Tetrahymena*. In some images, DNA is stained with TO-PRO-3. (**A–D**) Wildtype cells. A higher magnification of the cortical region boxed in A is shown in **B**; In panel **C**, the inset shows a higher magnification of a group of cilia of the boxed area. (**E–I**) CCTα-KO cells 26 hpm. **F** represents a higher magnification of a boxed region of the cell cortex shown in **E**. **H** and **I** are higher magnifications of boxed regions shown in **G**. Arrowheads in **F** and **H** show either shortening or absent TMs. (**J**–**M**) CCTδ-KO cells 26 hpm. **K** shows a higher magnification of an area boxed in **J**. Arrowheads in **K** show shortening TM bundles. (**M**) A differential interference image of a portion of CCTδ-KO cell. The arrow points at a branched ciliary tip. (**O–Q**) Anti-centrin staining of respectively WT, CCTα-KO and CCTδ-KO showing disorganization of ciliary rows in CCT depleted cells; (**Q**) shows a higher magnification of a boxed region from cell shown in (**P**), depleted from CCTα-KO, where it is observed a variation in the distance between two consecutive basal bodies and presence of gaps reflecting absence of basal bodies in the row. (**S**–**V**) Tubulin-KO cells 26 hpm stained with antibody directed to α-tubulin. In (**U**), higher magnification of area boxed in (**T**), and inset in V arrows point at branched ciliary tips. Scale bar represents 10 µm except if mentioned differently.

*Tetrahymena thermophila* cells, like most ciliates, have two nuclei, the germline, transcriptionally silent micronucleus (MIC) and the somatic, transcriptionally active macronucleus (MAC). Using DNA homologous recombination, we constructed heterokaryon strains with disruptions of either *CCTα* or *CCTδ* genes only in the micronucleus using a *neo2* gene cassette that confers resistance to paromomycin [24]. To study the consequences of gene disruptions, we allowed pairs of knockout heterokaryons to mate and produce progeny cells with new macronuclei developed from the zygotic micronuclei and expressing the gene knockout phenotype. While control wildtype strain matings produced viable conjugation progeny at the frequency of 95% (n = 200), no viable paromomycin-resistant progeny was recovered from matings of CCTα or CCTδ knockout heterokaryons (n = 180 and 107 respectively). Inspection of drop cultures containing isolated pairs of mating CCT (α or δ) heterokaryons revealed exconjugant cells that separated but failed to give rise to vigorous clones. These non-viable exconjugants were assumed to be progeny of mating heterokaryons that were expressing the CCT subunit knockout phenotypes. Typically these non-viable exconjugants presumably lacking a zygotic expression of either *CCTα or CCTδ* died after ∼50 hpm (hr post mixing of heterokaryons). Within this time, most of the CCTα and CCTδ heterokaryon progeny failed to divide even once and about 20% completed a single cell division. The progeny that had divided often produced two daughter cells unequal in size (data not shown). While at 26 hpm, progeny cells of a control cross had a nuclear organization typical of a vegetative cell (1 MIC and 1 MAC) most of the CCT heterokaryon progeny had the pattern of DNA typical of an early exconjugant cell (two MACs and one or two MICs, Figure 1E and L compare with wildtype in D), consistent with an arrest in cell differentiation at an early post-conjugation stage and failure to enter a vegetative cell cycle.

Similar observations were made for CCT knockout heterokaryon progeny that were isolated into MEPP medium that supports growth of cells lacking either a functional oral apparatus [25] or cilia [26], [27]. Thus the lethality of CCT heterokaryon progeny is not caused by loss-of-function of cilia or oral apparatus, both organelle types required for phagocytosis. All these observations indicate that both *CCTα* and *CCTδ* genes are essential.

### Cells lacking zygotic CCTαp or CCTδp loose cytoplasmic and cortical microtubules and have structural defects in axonemes

**Figure 2:**
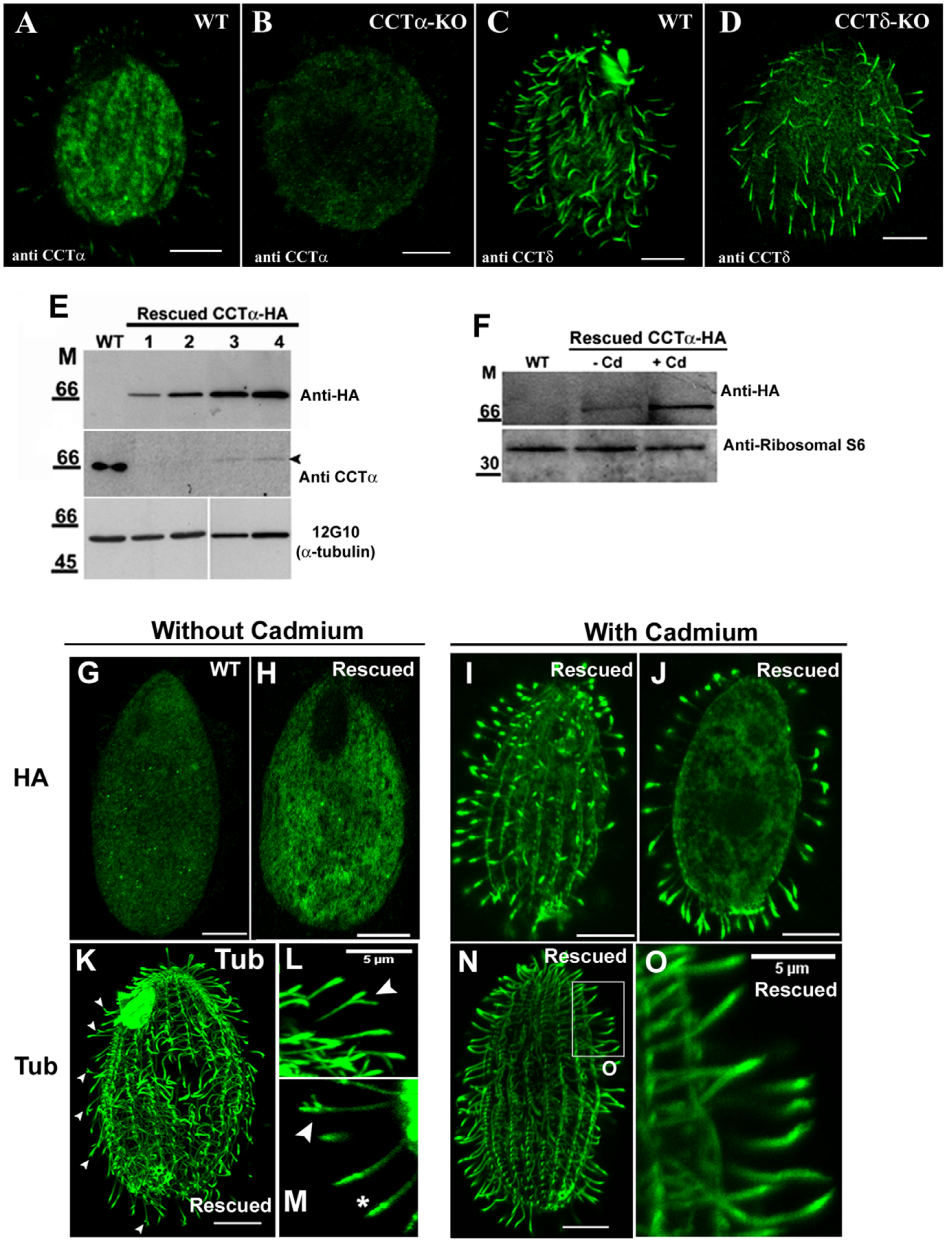
CCTαp-HA rescues CCTα-KO, localizes in cilia and its abundance affects cilia tips. **A–D**) Confocal immunofluorescence with antibody directed to CCTα (**A** and **B**) and CCTδ (**C** and **D**) in wildtype, CCTα-KO and CCTδ-KO *Tetrahymena* cells showing that depletion of CCT genes reduces CCT levels in cell body and cilia. All images were taken with exactly under the same exposure conditions. (**E**) Western blots of total proteins obtained from wildtype (WT) and rescued CCTα-KO cells expressing CCTαp-HA under cadmium-inducible promoter probed either with anti-HA, anti-CCTα (affinity-purified) antibodies, or the anti-α-tubulin 12G10 antibody. Lane 1 and 3- cells grown in SPPA with 1.5 µg/ml of cadmium chloride for 24 and 48 h, respectively; lane 2 and 4- cells grown in SPPA with 2.5 µg/ml of cadmium chloride for 24 and 48 h, respectively. (**F**) Western blots of total proteins of WT and rescued CCTαp-HA cells, grown in SPPCT medium in absence (−) or presence (+) of cadmium (Cd) probed with anti-HA and anti-ribosomal S6 antibodies. (**G**–**O**) Epitope-tagged CCTαp localizes to cilia and its levels affect the integrity of ciliary tips. *Tetrahymena* CCTα-KO cells were rescued by introduction of a transgene expressing CCTαp-HA under MTT1 promoter, grown in the absence or presence of cadmium, and processed for immunofluorescence using anti-HA (**G** to **J**) and anti-α-tubulin (12G10) (**K** to **O**) antibodies. (**G**) A wildtype cell stained with anti-HA antibody. (**H**–**J**) A CCTαp-HA-expressing rescued cells stained with anti-HA antibodies and grown in SPPCT medium for 76 h, without cadmium (**H**) and with 2.5 µg/ml of cadmium (**I** and **J**); **J** shows an internal optical section of the cell shown in **I**. (**K**–**O**) CCTαp-HA rescued cells stained by anti-α-tubulin antibody that were grown in SPPCT medium for 76 hr either without cadmium (**K**–**M**), or with 2.5 µg/ml of cadmium (**N**–**O**). (**L** and **M**) Higher magnification of ciliary regions of cells grown in SPPCT without cadmium to show the abnormal cilia tip. (**O**) A higher magnification of a boxed region from cell shown in (**N**). Scale bar = 10 µm, except if mentioned differently.

Next we analyzed the morphology of the non-viable progeny of mating CCT heterokaryons before their death. These cells were designated as CCTα-KO and CCTδ-KO. By immunofluorescence of the CCTα-KO and CCTδ-KO cells with antibodies that recognize respectively CCTα and CCTδ proteins, we observed a reduction of signal in the KO cells (Figure 2A–D). Typically CCTα-KO and CCTδ-KO cells were smaller and more rounded as compared to wildtype (Figure 1G, J, compare with A, C). Both the CCTα-KO and CCTδ-KO cells showed progressive loss of microtubules in the cell body (Figure 1E and J). At 26 hpm, in the CCTα-KO cells, the cortical longitudinal bundles (LM) and transverse microtubule bundles (TM) were less apparent based on immunofluorescence with an antibody against α-tubulin (Figure 1E–H, compare with A to C). It appears that in CCTα-KO cells, LMs are thinner, and TMs are shorter, suggesting shortening or loss of individual microtubules within the cortical bundles (Figure 1E–H, compare with A–C). At 36 hpm the LMs and TMs were no longer detectable in CCT-KO cells (data not shown). The intracytoplasmic microtubules were nearly completely absent at 26 hpm (Figure 1L, compare to 1D). The CCTα-KO and CCTδ-KO cells had fewer cilia, especially in the mid and posterior region of the cell (Figure 1G and J). In a normal cell, new cilia are inserted primarily within the mid and posterior segment of the cell. *Tetrahymena* cells assemble new basal bodies in an asymmetric pattern, primarily within the central and posterior region of the cell. The fact that the density of cilia decreases in the central and posterior portion of the cell indicates that CCT KO cells are unable to assemble new cilia but are able, at least for sometime, to maintain pre-existing cilia (that were assembled before the KO induction). In the CCT KO cells, the basal body rows revealed by anti-centrin antibodies were often distorted and tended to be further apart (Figure 1P–R compare with O). Gaps in the rows of basal bodies were apparent suggesting that the assembly of new basal bodies is also affected by CCT depletion (Figure 1R).

Cilia in the KO cells appeared shorter as compared to wildtype (exconjugant) cells. In the CCTδ-KO cells at 26 hpm, cilia had an average length of 5.00±0.56 µm, (n = 196) as compared to 6.77±0.59 µm in the wildtype (n = 175). Shorther cilia were also detected in CCTα-KO at 26 hpm, with an average length of 5.24±0.42 µm (n = 35), with similar values at 32 hpm (5.01±0.43 µm (n = 109)). These differences are statistically significant (t-test; p<0.001) (see graph Figure S1A). Strikingly, many cilia in CCT-KO cells had splayed tips (31% (n = 519) and 58% (n = 775) of CCTα-KO and CCTδ-KO cilia, respectively). The splayed segments measured on average ∼0.8 µm.

Since one of the major substrates of the cytosolic chaperonin CCT is tubulin, we compared the phenotypes of CCTα- and CCTδ-KO cells with the phenotype of cells entirely lacking zygotic expression of conventional α- and β-tubulin (products of *ATU1*, *BTU1* and *BTU2* genes). To this end, we mated heterokaryons that carry in their MICs disruptions of all conventional α-tubulin and β-tubulin genes, namely *ATU1*, *BTU1* and *BTU2* (J.G., unpublished results). As expected, no viable paromomycin-resistant progeny was obtained from crosses of tubulin knockout heterokaryons (n = 120). Typically exconjugants separated but failed to establish viable clones and died before 48 hpm. At ∼26 hpm, the tubulin-KO cells had a spherical shape and lacked most of LMs, TMs, and intracytoplasmic microtubules (Figure 1S, T and V) and had a dramatically reduced number of cilia. Despite the rapid loss of microtubular structures, some tubulin-KO exconjugants had divided once, in most cases asymmetrically. The tubulin-KO cells had fewer cilia (consistent with a failure of assembly of new units) and among the remaining (pre-exisiting) cilia 67% (n = 426) had splayed tips (Figure 1U and inset in V). The length of pre-existing cilia was slightly reduced at 26 hpm (5.87±0.54 µm (n = 102 cilia), with similar values at 36 hpm (5.65±0.47 µm (n = 112), (compare to wildtype cilia length, 6.77±0.59 µm (n = 175)). The differences are statistically significant (t-test; p<0.001) (Figure S1A). Thus, the consequences of loss of CCT subunits and loss of tubulin are similar except that the length of cilia is slightly more affected in the CCTδ-KO cells. These data argue that to a large extent, the consequences of loss of CCT activity could be mediated by lack of proper folding of cilia-destined tubulin by CCT.

### CCTα and CCTδ depleted cells are unable to reciliate

The capacity of the CCTα and CCTδ-KO cells to reciliate after deciliation was investigated using a deciliation protocol adapted to a small number of cells (see Materials and Methods S1 and References S1). We have used cells at ∼20 h of KO. The same procedure was performed in WT cells as control. Contrarily to WT cells, the CCTα and CCTδ-KO cells after 20 h of KO induction are unable to recover their cilia. Very few KO cells were able to reciliate, and in such cases, there was only a partial reciliation, with a random distribution of the new cilia (Figure S2) (data not shown for CCTα). This observation confirms the CCTα and CCTδ are required for assembly of new cilia.

### HA epitope-tagged CCTαp rescues CCTα-KO cells and localizes to cilia

To address the specificity of the observed CCT gene knockout phenotypes, we tested whether the progeny of mating CCT knockout heterokaryons cells could be rescued by reintroduction of a wildtype CCT gene fragment encompassing the disrupted region. To this end, we mated pairs of CCT heterokaryons, subjected them to biolistic bombardment using a corresponding CCT gene fragment that was designed to replace the disrupted CCT gene sequence by DNA homologous recombination (as described in Material and Methods), and selected progeny cells with paromomycin, to which resistance was conferred by the *neo2* cassette. In principle, we attempted to select surviving progeny that had replaced some of the disrupted copies of a CCT gene with wildtype copies in the new MAC. After biolistic transformation, for both CCTα and CCTδ mating heterokaryons, ∼97% of the wells (n = 480 corresponding to 10^7^ mating cells) contained drug-resistant growing cells while no such wells appeared in the same number of selected mock-transformed CCT mating heterokaryons. The presence of the CCT transgenes in the rescued cells was confirmed by PCR (Figure S3). Thus, we confirm that the lethality of CCT gene knockout mating heterokaryons is caused by disruption of CCT *loci*.

To test whether the lethality in progeny of CCT knockout heterokaryons is caused by a loss of the CCT subunit protein, and not solely by gene targeting, we attempted to rescue the mating CCTα heterokaryons by biolistic bombardment with a fragment that was designed to insert a gene encoding an HA-tagged CCTα under the control of the cadmium-dependent *MTT1* promoter into the non-essential *BTU1* locus [28]. Rescues were observed at the frequency of ∼92% of the wells (n = 480 corresponding to 10^7^ mating cells). The genomic DNA extracted from CCTα-HA rescued cells was found to contain the transgene fragment (Figure S4A and B). Using antibodies against the HA and CCTα -subunit we also confirmed by western blot that the rescued cells expressed CCTα p-HA protein (Figure 2E). As expected for a MTT1-driven transgene, the levels of CCTα-HA protein were increased with either the higher dose or longer exposure to cadmium (Figure 2E). Thus, we have successfully expressed a MTT1-driven copy of CCTα gene in cells that lack the endogenous CCTα gene. Interestingly, polyclonal antibodies that were generated against a CCTα peptide, reacted weakly with the (more slowly migrating) transgene protein in rescued cells as compared to wildtype protein in control cells (Figure 2E). Since the antibodies were generated against the last 12 amino acids of CCTα [29], addition of HA to the C-terminus could have a steric effect on the epitopes of the polyclonal antibodies. In absence of exogenous cadmium, CCTαp-HA was localized primarily to the cell body and was not detected in cilia (Figure 2H compare with negative control in 2G). When cadmium chloride (2.5 µg/ml) was added to the medium for 76 h, a stronger signal of CCTαp-HA was detected and the protein was prominently present in cilia and accumulated at the ciliary tips (Figure 2I and J). Next, we investigated the consequences of lowering the levels of CCTαp-HA expression, by growing rescued cells without exogenous cadmium (in an SPP medium from which the residual cadmium ions were removed by exposure to chelex-100 resin referred as SPPCT, see Material and Methods). Wildtype cells had similar growth rates in SPPCT supplement with exogenous cadmium to the growth shown in SPPCT without addition of cadmium. On the other hand, the rescued CCTα-HA cells had a growth rate slightly lower when grown in SPPCT without cadmium than in SPPCT complemented with cadmium (data not shown). It is worth to mention that while lack of exogenous cadmium has resulted in a dramatic decrease in the levels of CCTαp-HA, small amount of the protein was still present, likely because the MTT1 promoter has a non-induced basal level of expression, mimicking a knockdown of CCTα (Figure 2F). Strikingly, cilia were shorter in cells with reduced levels of CCTαp-HA (grown without cadmium), than in wildtype cells grown under the same conditions (Figure S1B). Furthermore, based on immunofluorescence with an antibody against tubulin, these cells have an increased number of cilia with splayed tips or abnormal spotted tubulin staining pattern at the tips (Figure 2L and M, compare with O). Also, these phenotypes were not observed in wildtype cells growing in SPPCT (data not shown).

To conclude, we observed that CCTαp-HA, when moderately overexpressed localizes to cilia and is enriched at the tips. These data are consistent with our previous observations [16] that CCTα is a ciliary protein. This localization was also confirmed by isolation and fractionation analysis of cilia obtained from wildtype cells (see Materials and Methods S2 and References S2). The serum antibody anti CCTα used in these experiments recognized specifically one band in both axonemal and membranar fractions (Figure S5A). To assess the effectiveness of fractionation, we re-probed the same blot with anti α-tubulin antibody. As expected, tubulin, the major protein of the axonemes was weakly detected in the membrane fraction (Figure S5A). The specificity of the antibody was tested by peptide pre-absorption to the antibody as shown in Figure S5B. Moreover, our results show that the depletion of CCTα affects the structure of axoneme tips.

### The G346E mutation in *Tetrahymena* CCTαp leads to a temperature-sensitive growth and affects the function of oral cilia

We took advantage of the availability of CCTα knockout heterokaryons to introduce a mutation into CCTαp that could affect cilia. Kim and colleagues [13] showed that BBS6, a protein whose mutation causes a ciliopathy, the Bardet-Biedl Syndrome, has amino acid sequence homology with CCTα. The genome of *Tetrahymena* and many other non-vertebrate eukaryotes lacks an obvious BBS6 sequence. These observations suggest that BBS6 is a vertebrate-specific variant of CCTα that has evolved cilia-specific functions. Consequently, organisms like *Tetrahymena* that lack BBS6, could be using CCTα for ciliary functions, as is supported by our data so far. To identify amino acids of CCTα that could be important in the context of cilia, we produced a multiple sequence alignment of BBS6 and CCTα proteins from a few ciliated organisms. Overall, the BBS6 and CCTα sequences are 19% identical (30% of similar) (Figure S6A). We examined amino acids that represent the apical domain of CCTα and could contribute to the substrate-binding site [30]. Within this domain, one amino acid is conserved between BBS6 and CCTα of diverse organisms: glycine 346 from *T. thermophila* CCTα. Importantly, in humans, a mutation at the corresponding position, G345E, causes Bardet-Biedl Syndrome [13]. Since the mutation occurs in the CCTα apical domain (Figure S6B) we hypothesised that the mutation G346E could interfere in the interaction between CCTα and folded substrates relevant to cilia assembly/maintenance. To investigate the impact of the mutation in the native functional structure of the CCTα protein, we predicted with the ProModII program [31] and compared the secondary structure of the wildtype and mutated G346E CCTα apical domain (Figure S6C). The model of the partial CCTα structure obtained was based on the crystal structure of the subunit α of the chaperonin thermosome from *Thermoplasma acidophilum* [32]. We observed that the replacement of the glycine for a glutamate has led to disruption/disappearance of several α-sheets (see arrow in Figure S6C) which might interfere with the flexibility of this domain that is required for folding. Indeed, the apical domain contains a helical protrusion [18], which is involved in opening and closing the central cavity of the chaperonin. The remnant of the secondary structure of this apical domain did not suffer any change with the referred substitution.

We used a fragment encoding a CCTα with the ciliopathy-based mutation, G346E, in an attempt to rescue mating CCTα heterokaryons. Besides the single mutation, the fragment encoded an otherwise wildtype sequence and was intended to replace the disrupted sequence at the native locus. Rescued cells were isolated, indicating that G346E is not a lethal mutation. The genomic DNA of these transformants was analyzed by PCR and sequenced, revealing the presence of two products corresponding to the neo-disrupted and the introduced G346E encoding CCTα allele (Figure S4C).

The G346E mutant cells grew extremely slowly on the regular SPP medium. Furthermore, the CCTα-G346E cells were temperature-sensitive, growing more slowly at 30°C as compared to 16°C. At 16°C, the G346E population contained mostly normal-looking cells in respect to size and shape, but some of these cells displayed erratic movement patterns including prolonged periods of spinning around the antero-posterior axis (results not shown). At 30°C, 50% of G436E cells had a normal shape (average dimensions 25×47 µm, n = 488), 27% were extremely elongated (average dimensions 69×50 µm, n = 260), 11% had a drop-like shape (n = 105) and 13% were very large so called monster cells (90×60 µm to 50×45 µm, n = 127).

**Figure 3:**
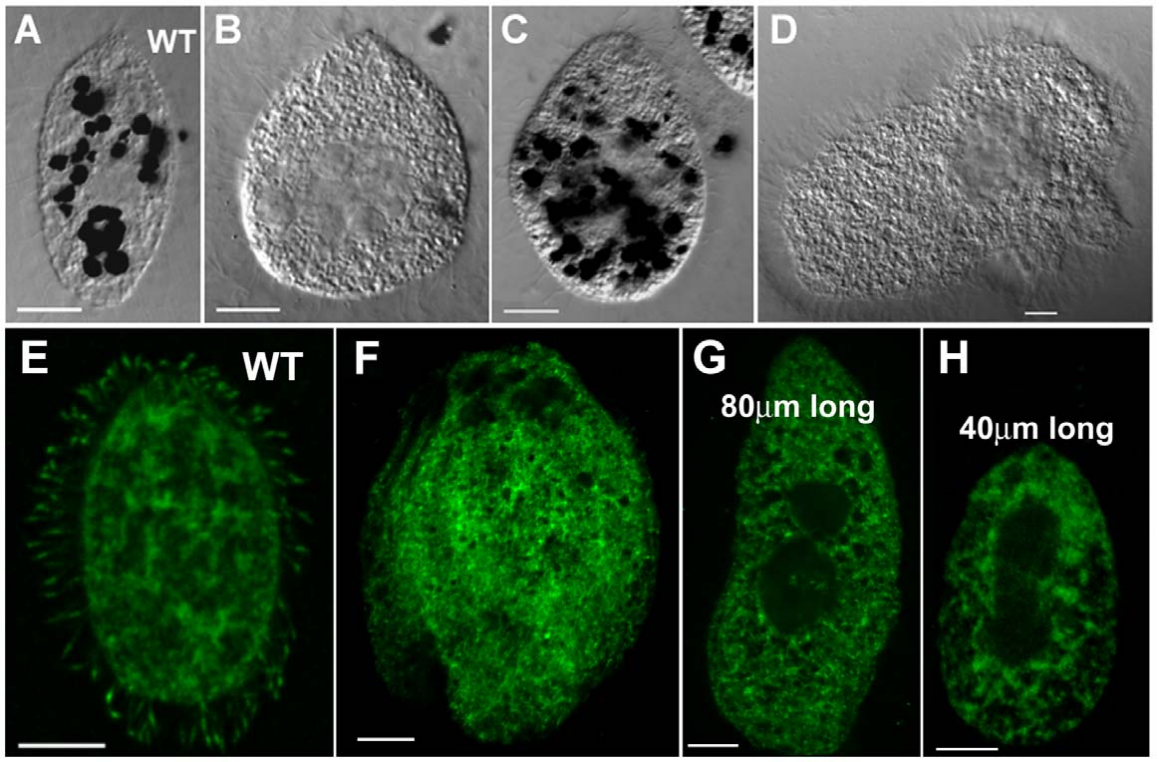
Absence of CCTα in cilia of CCTαG346E mutant cells correlates with abnormal cilia tips and disfunction of oral cilia. (**A** to **D**) Phagocytosis capacity of CCTα-mutG346E strain was evaluated by exposition of cells to Indian ink. (**A**) Wildtype cell used as control; (**B** to **D**) CCTα-mutG346E cells exposed to Indian ink; (**B**) and (**D**) were unable to form food vacuoles and are void of black granules (n = 1448). (**E** to **H**) *Tetrahymena* wildtype (**E**) and CCTαG346E mutant (**F** to **H**) cells were processed for immunofluorescence using CCTα (affinity-purified) antibodies. Wildtype cells show CCTα localization in cilia while in cells carrying the CCTα-G346E mutation it is noticed the absence of CCTα signal in cilia. The images were taken with exactly the same settings of gain and contrast. Scale bar  = 10 µm.

We noticed that these cells grew better in MEPP media that stimulates the uptake of nutrients by pathways that do not require phagocytosis in the oral apparatus [25]. We tested their capacity of performing phagocytosis vacuoles adding Indian ink to the medium and quantified the cells that presented food vacuoles containing ink (Figure 3A–D). Ninety four percent of G436E cells (n = 1448) were unable to uptake ink. Thus, oral cilia may not be fully functional in the G436E strain. Noticeable, the 6% of the mutant cells that were able to ingest ink, and that were designated by “normal looking cells” started to prevail in the culture when mutant cells grew at 30°C for long periods (several weeks). This led us to investigate if the introduced mutation in the CCTα coding region gene was still present in these cells. By sequencing analysis we confirmed that the mutation G346E continued to be present in the CCTα gene sequence of these cells, being the only allelic form of CCTα found. Indeed, the mutation G346E was inserted in the region of CCTα coding region that have been removed when constructing the KO heterokaryons strains. Therefore, since these were the cells that were rescued by the introduction of the G346E mutated gene, the observed recovered phenotype could never be a consequence of a recombination event between the wild type CCTα gene and the mutated one. Most probably, these cells constitute a suppressor strain where a second mutation occurred restoring the original phenotype, by reverting the effect of CCTαG346E mutation, and are under natural selection when growth occur over long periods.

**Figure 4:**
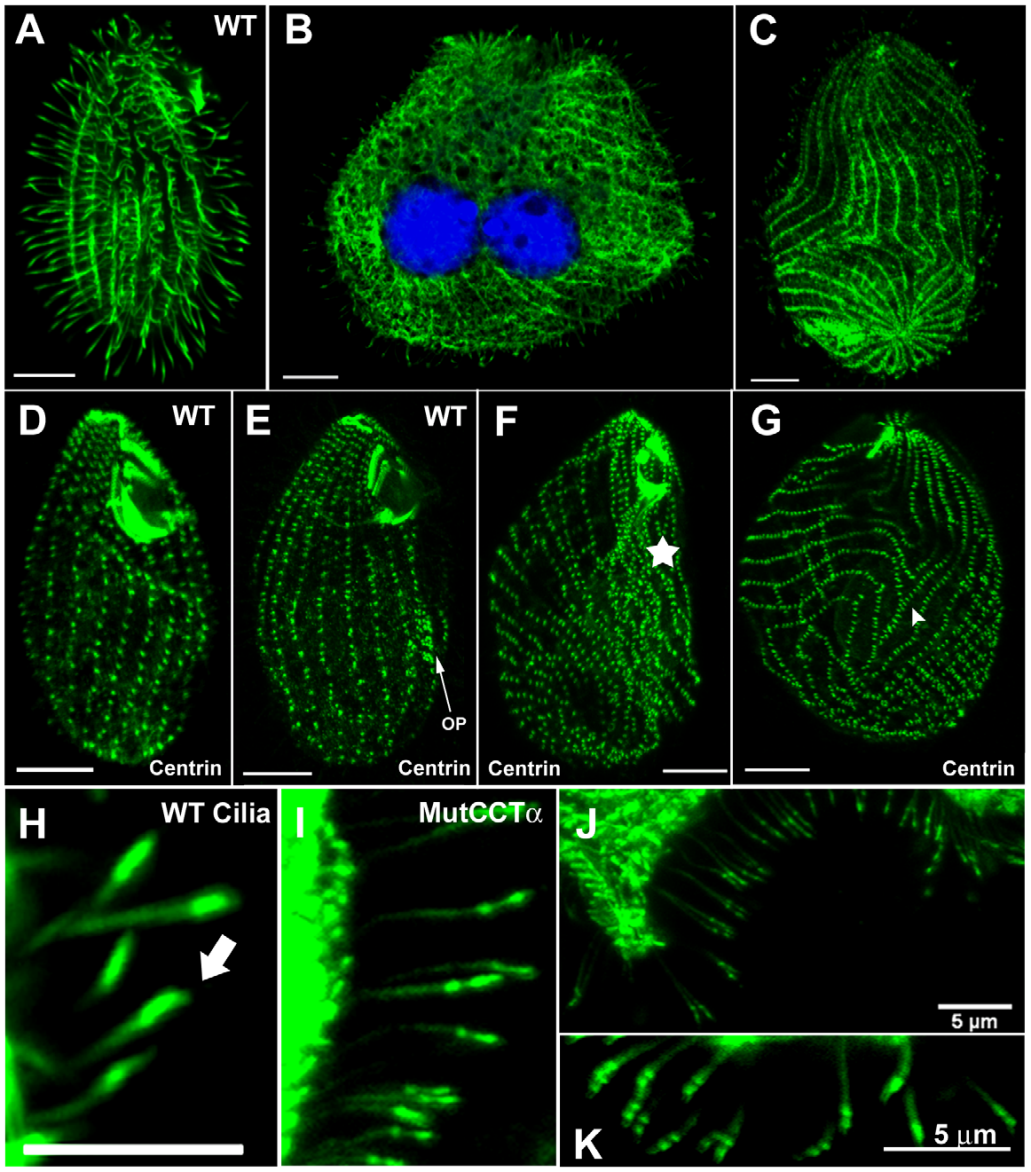
CCTα G346E mutation leads to severe cortical and ciliary defects. Indirect immunolocalization of tubulin and centrin in *Tetrahymena* wildtype (WT) and CCTα-mutG346E cells. These cells were processed for confocal microscopy analysis using the antibodies directed to α-tubulin (**A** to **C** and **H** to **K**) or centrin (**D** to **G**). DNA was stained with TO-PRO-3. (**B**) Merged image resulting from the superimposition of the image obtained for TO-PRO-3 and the corresponding image for α-tubulin showing CCTα-mutG346E-containing cell with impaired cytokinesis; note that the cell has already divided the MAC and has a double width comparing to wildtype cell (**A**). (**C**, **F** and **G**) CCTα-mutG346E cells with abnormal shape and width; note the misalignment of ciliary rows in relation to the longitudinal axis; also note the intense and disorganized region of basal bodies above the oral primordium (OP) (five-star); compare with OP found in WT cells (**E**). (**H**) Higher magnification of WT cilia tips (arrow). (**I**–**K**) Higher magnification of cilia from CCTα-mutG346E cells to emphasize cilia defects. Scale bar  = 10 µm except when indicated differently.

The microtubule cytoskeleton of CCTα-mutG346E-carrying cells was analyzed by immunofluorescence using an antibody against α-tubulin (Figure 4). Mutant cells frequently contained multiple sets of nuclei and multiple cortical domains, *e.g* oral apparatus, and constitute the typically designated monster cells (Figure 4B and C compare with wildtype in A), consistent with failures to undergo cytokinesis. However the normal-looking cells were able to divide completely (data not shown). The evident defects in completing cytokinesis and their multiple attempts to divide led mutant cells to exhibit dramatic alterations in the organization of ciliary rows as confirmed using an antibody against centrin (Figure 4F and G, compare with D and E). In contrast to wildtype cilia (Figure 4H) that have a spear-like staining of tubulin at the tip, the mutant cells showed an abnormal staining at the most distal part revealed as a strong spotted staining of tubulin (Figure 4I to K). Since low levels of CCTα lead to abnormalities of cilia tips (Figure 2L and M) and mutant cells also have abnormal tips we decided to investigate if the mutated form of CCTα was targeted to cilia. We observed that the antibody against CCTα did not give any ciliary staining in the mutant cells, even when the body of the cell is clearly labeled (Figure 3 F–H compare with E).

Taken together these data clearly show that the CCTα mutation G346E affects CCTα cilia localization which in turns affects cilia tips. The observed effect of the CCTα mutation G346E supports the previous evidences shown in this paper that CCTα is required for cilia structure maintenance, particularly at the tip level.

## Discussion

We have investigated the function of CCTα and CCTδ subunits of the eukaryotic cytosolic chaperonin in *T. thermophila*. To our knowledge, this is the first functional study of CCT subunits in a ciliated model. We show that the *CCT*α and *CCT*δ genes are essential in *T. thermophila*, as shown earlier in yeast [33], [34]. The essential function of CCT is not unexpected since the CCT complex participates in the folding of essential cytoskeletal proteins (actin and tubulin). Also CCT may mediate the folding of 1000–2000 other proteins that play diverse and critical functions in the cell, as, for example, cell cycle progression, chromatin remodeling, assembly of nuclear pore complex and protein degradation [35], [36].

The phenotypes of cells lacking either CCT subunits or tubulin are quite similar: these cells fail to grow within 1–2 generations, loose cytoplasmic and cortical microtubules, fail to assemble new cilia and have defects at the tips of microtubules in pre-existing axonemes. Thus, it is possible that to a large extent, the lethality induced by CCT subunit loss-of-function is caused by failure to fold tubulin. Consistently, in mammalian cultured cells, reduction of CCT levels by 90% (due to siRNA-mediated knockdown) strongly reduced the levels of total and newly synthesized α- and β-tubulin [37]. The observed splayed tips of axonemal microtubules could be explained by increased curvature of protofilaments that depolymerize [38]. It is likely that tips of axonemes are unstable due to lack of addition of new tubulin subunits. The simplest explanation of our observations is that proteins destined to cilia, including tubulin, requires folding by CCT.

Despite the fact that the phenotypes of CCT subunit loss-of-function can be explained by the resulting failure in tubulin folding in the cell body, published work and some data presented here continue to support a role for CCT subunits inside cilia. Thus, expression of CCT subunit genes is increased during cilia regeneration in *Tetrahymena* [14] and *Chlamydomonas* [39]. While this result alone could be explained by a cell body-restricted activity whose levels increase during ciliation, localization and proteomic studies have detected some CCT proteins in cilia and centrioles/basal bodies in *Tetrahymena*, sea urchin embryos and *Chlamydomonas* [9], [16], [40]. Here we present complementary data showing that CCTα is present in both membrane/matrix and axonemal fractions of cilia (see Figure S5), suggesting that the protein is interacting with the axonemal microtubules while circulating in the ciliary compartment. Also the CCT-depleted cells show a reduced level of this protein in cilia (see Figure 2). Moreover, we show that the epitope-tagged CCTα which rescues the gene knockout lethal phenotype localizes to cilia. Thus, either the folding activity of CCT chaperonin also occurs inside cilia, or the CCT subunits found in the ciliary compartment have other functions. Interestingly, other chaperones not required for tubulin folding have been found inside cilia. For example, Hsp70 and Hsp90 were found in *Tetrahymena* cilia [10] and Hsp70 was detected in *Chlamydomonas* flagella and ciliated cells of sea urchin embryos [7], [41]. Hsp70 was identified as one of the components of a 17S complex p28-containing inner dynein arms in *Chlamydomonas* [42]. Noteworthy, both Hsp70 in *Chlamydomonas* [7], and the epitope-tagged CCTα in *Tetrahymena* (this study) preferentially localize to the tips of assembled cilia. Since ciliary proteins are subjected to significant mechanical stress, their function may require a relatively high level of turnover to replace damaged proteins. Inside cilia, molecular chaperones could be involved in quality control and turnover of ciliary proteins. In agreement with this model, in *Chlamydomonas*, Hsp70 and Hsp40 affect flagellar movement possibly by maintaining/transforming protein conformations [12], [43]. CCT could be required for the maintenance of axonemal proteins subunits such as tubulin and ciliary actin [44], [45], or alternatively, for their assembly and/or turnover. This hypothesis is supported by our observations that show both the localization of overexpressed CCT at the tips of cilia, as well as defects of ciliary tips in cells depleted in CCT activity. Importantly, in *Xenopus* multi-ciliated cells, CCTα and CCTε were localized in punctuate structures along the ciliary axonemes, and their mislocalization induced by the depletion of an antagonist of Wnt pathway (Fritz) has been correlated with fewer and shorter cilia phenotype [46]. Moreover, the fact that CCTδ-KO cells were unable to reciliate indicates CCT activity is important for new assembly of cilia, and this role may not be simply the cytosolic supplier of tubulin. Interestingly, *Tetrahymena* cells grown in an enriched medium and treated with cycloheximide can partially regrow cilia after deciliation suggesting the presence of a pool of stored tubulin [47] that cells could use for assembly of new cilia.

It is known the distal ends of axonemal microtubules are covered by caps, structures that connect axonemal microtubules to the cilia membrane [48], [49]. These structures were suggested to be involved in the assembly and maintenance of cilia, possibly regulating the assembly and disassembly of axonemal microtubules [50], [51], [52]. We can speculate that CCT subunits are associated with either the distal ends of axonemal microtubules or with caps. It is known that the CCT subunits γ, α, ζ and δ bind to *in vitro* assembled microtubules, and thus behave like microtubule-associated proteins (MAPs) [53]. Interestingly, CCT subunits bind to F-actin and reduce the filament elongation rate at the plus end in erythrocyte membrane cytoskeletons [54]. It is conceivable that through the ability to behave as end-binding MAPs, CCT subunits affect the assembly and turnover of tubulin on axonemal microtubules known to occur preferentially at the distal end of axonemes [55]. Additionally, CCTs may be involved in interactions between microtubules and the cilia membrane at ciliary tips. There is some evidence that CCT subunits interact with membranes. The adrenal medullary form of CCT (chromobindin A) efficiently binds to chromaffin granule membranes [56]. In human erythrocytes, CCTα is translocated to the plasma membrane following a heat-shock, interacting with the specialized membrane skeleton [57].

We show that CCTα-G346E mutation impairs CCTαp localization at cilia tips and those cilia present an abnormal pattern of staining with anti α-tubulin. These observations support a model that this CCT subunit has a direct ciliary role. As the evolutionary related BBS6 [13], CCTα may have a role in assembly of some complexes at cilia tips. Nachury and collaborators [58] have shown that the BBSome, an oligomeric complex of BBS (BBS1-2, BBS4-5, BBS7-9) proteins, was directly implicated in ciliogenesis by promoting vesicle trafficking to the cilia membrane. Very recently, it was shown that BBS6 forms with the other chaperonin-like BBS10 and BBS12 proteins (vertebrate-specific BBS genes), a complex with CCT proteins (CCT1-5 and CCT8) that is required for BBSome assembly [59]. Similarly to *Tetrahymena* CCTα depleted cells and CCTα-G346E mutant where oral and somatic cilia presented functional failures, the respiratory tract cilia of BBS6-/- mice showed structural abnormalities accompanied by functional defects affecting cilia tips and reduction of ciliary beat frequency [60]. Therefore, is tempting to suggest that in *Tetrahymena* CCT chaperonin does not require BBS6 to interact with BBSome subunits since CCTα evolutionary could be seen as its representative/substitute.

In conclusion, the construction of *Tetrahymena* CCTα- and CCTδ-KO strains has helped to define the role of CCT subunits in a ciliated organism. We show in this study that CCT subunits are needed for assembly of cilia and maintenance of axoneme structure, especially at the tips of cilia.

## Materials and Methods

### Cells and culture conditions

Strains used in this study are listed in Table S1. *Tetrahymena* cultures were grown in SPP [61] supplemented with an antibiotic/antimycotic mixture at 100 U/ml penicillin, 100 µg/ml streptomycin, and 0.25 µg/ml amphotericin B. In some experiments we used the MEPP medium on which *Tetrahymena* cells grow in the absence of phagocytosis [25]. The SPPCT (metal-depleted medium; D. Dave and J.G, unpublished) was used in some experiments. SPPCT was prepared by depleting the SPP medium from ions with 5% of Chelex-100 beads (BioRad) followed by complementation with trace metals (100-fold concentrated: 170 mM Co(NO_3_)_2_.6H_2_O; 0.71 M MnSO_4_.H_2_O; 6.8 M CaCl_2_.2H_2_O; 9M EDTA ferric sodium salt (C_10_H_12_N_2_NaFeO_8_); 200 M MgSO_4_.7H_2_O).

To assay phagocytosis, India ink was added at a final concentration of 1%, cells were incubated for 30 min and were scored for the presence of food vacuoles (filled with black ink).

### Germline disruption of CCTα or CCTδ genes

To disrupt either *CCT*α or *CCT*δ genes in the MIC, we introduced *neo2* cassette-interrupted targeting fragments into early mating cells using a biolistic gun and produced heterozygous transformants as described [62], [63]. To prepare a targeting plasmid for disruption of the *CCTα* gene, a ∼1.6-kb genomic fragment that included ∼400-bp of the 5′UTR plus the first 1.2-kb of the coding region of *CCTα* (including the translation initiation codon), was amplified with primers Alf-5F and Alf-5R containing restriction sites for *Sac*II and *Bam*HI respectively (primers Alf-5F:5′-TCCCCGCGGATGAATGAAAGAGTGAGATG-3′ and Alf-5R:5′-CGCGGATCCTTCAACAGCATCAACAACGA-3′). This fragment was cloned into the plasmid p4T2-1 [24], a *neo2* cassette plasmid. The resulting plasmid was digested with *Cla*I and *Xho*I and used to insert a ∼1.2-kb of 3′ UTR of *CCTα*, with the last 813-bp of genomic fragment of *CCTα*, including the codon stop. This fragment was amplified with the primers Alf-3F and Alf-3R containing the restriction site of *Cla*I and *Xho*I respectively in their flanking regions, (primers Alf-3F:5′-CCATCGATGAATGTGCTGAAGTTTACGA-3′ and Alf-3R: 5′-CGGCTCGAGCCCATTCTACATCTTATCC -3′), to create the plasmid pNeo2CCTα.

To prepare a plasmid for the disruption of the *CCTδ* gene, a 262-bp of 5′UTR, with the initial ∼1.6-kb genomic fragment of *CCTδ* including the first codon, was amplified with addition of SacII and *Bam*HI sites in the primers respectively (primers Delt-5F:5′-TCCCCGCGGTATGAATTGTTTTGAAGTGT-3′ and Delt-5R: 5′- CGCGGATCCTCAAT- CAATTCAGTGTCTTC-3′). This fragment was cloned into p4T2-1 using *Sac*II and *Bam*HI sites. The resulting plasmid was digested with *Cla*I and *Xho*I and used to insert a ∼1.5-kb of 3′ UTR of *CCTδ*, with the last 364-bp of the genomic fragment of *CCTδ*, including the stop codon. This fragment was amplified with the primers containing the restriction site of *Cla*I and *Xho*I respectively in their flanking regions, (primers Delt-3F: 5′-CCATCGATGACTAG- AGAAATGAAGGGTGTT-3′ and Delt-3R: 5′-CGGCTCGAGTAAGAAGACTGTTGA- TACCG-3′), to create pNeo2CCTδ.

For germline targeting, each disruption plasmid (pNeo2CCTα and pNeo2CCTδ) was digested with *Sac*II and *Xho*I and used to transform mating CU428.1 and B2086.1 strains by biolistic bombardment. For each transformation, approximately 10 µg of DNA was used to coat gold bombardment particles of 0.6 µm in size (Bio-Rad). Gene replacements mediated by these targeting fragments were designed to remove ∼800-bp of regions encoding highly conserved domains of the CCTα and CCTδ proteins. Heterokaryons were generated by bringing the micronucleus to homozygosity using a star cross while allowing the disrupted alleles to assort from the macronucleus [62].

### Rescues of mating CCT knockout heterokaryons with tagged and mutated CCT-encoding transgenes

To test whether the lethality associated with disruption of the CCTα and CCTδ is caused by loss-of-function of these genes, we attempted to rescue mating knockout heterokaryon cells with corresponding fragments of DNA containing the coding sequence of CCTα and CCTδ genes, respectively. The genomic fragment of CCTα gene was obtained by PCR with the primers Alf-5F: 5′-TCCCCGCGGATGAATGAAAGAGTGAGATG-3′ and Alf-3R: 5′-CGGCTCGAGCCCATTCTACATCTTATCC -3′, and cloned into T-Vector (Promega). In the case of the CCTδ gene, the fragment to clone was amplified with the primers Delt-5F (5′- TCCCCGCGGTATGAATTGTTTTGAAGTGT-3′) and Delt-3R (5′- CGGCTCGAGTAAG- AAGACTGTTGATACCG -3′) and digested after with *Sac*I and *Xho*I enzymes for biolistic transformation.

To create the CCTα G346E mutant strain we performed a somatic rescue transformation of CCTα-KO cells with mutated CCTα gene fragment obtained by site-directed mutagenesis [64] with an oligonucleotide: 5′-GAAGCTTCCTATCTAGAAGAAT- GTGCTGAAGTT-3′. In all the cases the biolistic transformation and selection of cells were performed as already described [65], [66]. The presence of the desired mutation in the CCTα gene of the transformed and rescued CCTα-KO cells was confirmed by PCR, using standard conditions, and analysis of the pattern obtained by restriction enzyme hydrolysis of the PCR products. It was also confirmed by sequencing the entire CCTα gene that no other modification was present.

To express CCTα-HA protein at levels comparable to physiological conditions, we rescued mating *CCTα* heterokaryon progeny, by introducing a fragment of DNA containing the coding sequence of CCTα-HA, without applying any selective pressure to increase the transgene copy number [66]. The transforming DNA was inserted by homologous recombination in an ectopic locus, the β-tubulin locus *BTU1*, and its expression was under the promoter MTT1 (metallothionein 1 protein), dependent of cadmium chloride. The *CCTα* KO heterokaryons strains (CCTA-A1 and CCTA-B5) were allowed to complete conjugation that takes approximately 14 h. Then, 24 h after mixing the heterokaryons (hpm, hours post mixing), the cells were transformed biolistically with the BTU1-MTT1-CCTα-HA-BTU1 cloned fragment. Transformants that integrated the transgene into the *BTU1* locus were selected with paromomycin (90 µg/ml) and cadmium chloride (1.5 µg/ml or 2.5 µg/ml).

### Indirect immunofluorescence microscopy

For staining KO cells, ∼50–100 cells were isolated into 10 µl of 10 mM Tris, pH 7.5, on a coverslip previously coated with poly-L-lysine (Sigma). These cells were generally isolated after 18 hpm that is ∼4 h after end of conjugation and consequently should be ∼4 h of KO. Coverslips were processed for immunofluorescence labeling as described in Thazhath and co-workers [67]. TO-PRO-3 (Molecular Probes) was used (1∶1000) to stain DNA during 90 min, at room temperature. The following primary antibodies were used: mouse 20H5 anti-centrin (1∶100, gift of Dr. Salisbury, Mayo Clinic, Rochester, MN), mouse 12G10 anti α-tubulin (1∶10, from University of Iowa, Developmental Studies Hybridoma Bank), rat purified (by affinity column) anti-CCTα (1∶10) (this work), crude rat serum anti-CCTα (1∶50) [29] and crude rat serum anti-CCTδ (1∶30) [16]. Secondary antibodies were goat anti-mouse Alexa 488 (Molecular Probes) (1∶500), goat anti-rat-FITC and goat anti-mouse-TRIC (Sigma) conjugates, both used at dilution of 1∶600. For immunolocalization of CCTα-HA protein in KO rescued cells, they were grown in falcon tube overnight without any drug except cadmium chloride, when added, washed, fixed and processed for immunofluorescence as the other slides. The primary antibody used was the mouse monoclonal anti-HA (Sigma) and the secondary antibody was goat anti-mouse Alexa 488 (Molecular Probes), in a dilution of 1∶500.

Cells were viewed using a Leica® TCS SP2 spectral confocal microscope (using 63x oil immersion with 1.40 NA). Images were assembled using Image NIH Image J. and Adobe Photoshop 6.0® software. The length of axonemes either on cells or isolated was determined on Z-project of confocal sections using NIH Image J.

### Protein electrophoresis and western blotting

To analyze the expression of the tagged CCTα-HA protein in the rescued cells, total protein extracts from 25000 cells were prepared, as well for wildtype cells, and used per lane. Briefly, cells were pelleted by centrifugation at 1600×g for 3 min, suspended in 1 ml of 10 mM Tris-HCl, pH 7.5 and further concentrated into a dense pellet by centrifugation at 1600×g for 3 minutes. Cell pellets were resuspended in 10 µl of 10 mM Tris-HCl, pH 7.5 and lysed with same volume of lysing buffer (62.5 mM Tris, pH 6.8, 2% SDS, 10% glycerol, 0.0005% Bromophenol Blue, 5% β-mercaptoethanol, final concentrations). Protease inhibitors were added at a final concentration of 0.5 µg/ml leupeptin, 10 µg/ml chymostatin, 10 µg/ml trans-epoxysuccinyl-L-leucylamido-(4guanidino) butane (E-64), and 15 µg/ml antipain. The mixture was boiled for 3 min at 95°C.

Electrophoresis and western blot analysis SDS-PAGE (10% (w/v)) gels were carried out as described elsewhere [29]. The following primary antibodies were used: rat polyclonal anti-CCTα (affinity-purified) (1∶500 dilution); mouse monoclonal anti-HA (1∶100 dilution) (Sigma) and rabbit anti-ribosomal S6 (1∶300 dilution) (Santa Cruz). Secondary antibodies used: peroxidase-conjugated goat anti-rat IgG (H+L) (1∶3000 dilution), goat anti-mouse IgG (H+L) (1∶4000 dilution) (Jackson ImmunoResearch Inc.) and goat anti-rabbit IgG (H+L) (1∶1000 of dilution) (Zymed). The protein molecular mass markers (mixture of proteins from 97 to 14 kDa) were purchased from Amersham Biosciences.

### Sequence analysis

For the multiple sequence alignment of CCTα and BBS6 protein sequences, the sequences obtained from NCBI and TIGR database and listed on Table S2 were used. The multiple sequence alignment was produced using the T-Coffee method [68] more appropriate for alignment of proteins with low percentage of identity as BBS6 and CCTα. The alignment was edited with GeneDoc program.

Prediction of the secondary structure of CCTα apical domain in wildtype and in the G346E mutant cells was done using the program ProModII [31], since the inserted mutation was in this protein domain. The model of the partial CCTα structure obtained was based on the coordinates of the subunit α of the chaperonin thermosome from *T. acidphilum* (Pubmed accession numbers: 1a6e, 1a6d and 1q2v). The visualization of the predicted structure is made by Rasmol program.

### Statistical analyses

The experiments were performed at least three times and the results were expressed as means ± S.D. Differences between the data were tested for statistical significance by t-test. P values less than 0.05 or 0.001 were considered statistically significant.

## Supporting Information

Figure S1 Cilia length in cells with low levels of CCTα and CCTδ. A) Cilia length in a population of wildtype, CCTα and δ KOs, or tubulin depleted cells were measured after 26 hpm. Cilia are significantly shorter in the absence of the referred CCT subunits, with p<0.0001. B) Cilia length in a population of wild type cells or in a population of rescued CCTα-HA cells, grown in medium with or without cadmium, was measured. Cilia are significantly shorter in rescued CCTα-HA cells grown without cadmium, with p<0,025, which correlates with the absence of CCTα in cilia (see Fig. 2H). Number of measured cilia was 145 in wildtype cells, n = 196 in CCTδ-KO cells, n = 35 in CCTα-KO (with similar values at 36 hpm with n = 112), n = 102 in tubulin KO cells, n = 90 in CCTα-HA cells growing with cadmium and n = 83 cilia in CCTα-HA cells growing without cadmium.
(0.07 MB TIF)

Figure S2 CCTδ depleted cells are unable to reciliate. A) Confocal immunofluorescence of αtubulin (using 12G10 monoclonal antibody) in *Tetrahymena* wildtype (WT) and CCTδ KO cells to analyze their reciliation capacity. (A and D) Non-deciliated cells (WT) and CCTδ KO cell. (B and E) Cells analyzed immediately after deciliation (R0); (C and F) Cells analyzed after 2 h of reciliation (R2h). WT cells are able to full reciliate and recover their swimming capacity (not shown), while CCT depleted cells are mostly unable to reciliate their cilia, or randomly recover a few cilia. Note the apparent gaps in transversal microtubules present in the CCT-KO cell. Scale bar  = 10 µm.
(1.87 MB TIF)

Figure S3 PCR analysis of strains obtained in rescue experiments to confirm their genotype. A) Analysis by PCR of the CCTα locus in wildtype cells and rescued CCTα-KO cells. For WT strain it was observed only one band (white arrowhead) corresponding to WT allele, whereas in rescued CCTα KO strain (RA+) an additional band (asterisk) corresponding to the disrupted-allele CCTα is visible. To facilitate the interpretation of the bands pattern a heterozygous strain for CCTα disruption was obtained by a cross of one of the CCTα-neo-disrupted heterokaryon strains with a WT strain. In the heterozygous (HZ) two bands were found, one corresponding to the WT allele (2.8 kb) and the other corresponding to the disrupted-allele of CCTα, with the expected size (3.5 kb). B) Analysis by PCR of the CCTδ locus in WT cells and in the rescued CCTδ-KO cells. Also, PCR analysis revealed two bands in rescued CCTδ-KO strain (RD+) confirming the presence of the WT and the disrupted allele.
(0.09 MB TIF)

Figure S4 Genotypic analysis of the CCTα-KO cells rescued with a HA tagged CCTα cDNA or genomic CCTαmutG346E. A) PCR analysis using genomic DNA from the rescued CCTα-HA strain (RAHA) with the: 1. pair of primers that amplify full cDNA CCTα; 2. Primer-F for initiation codon of CCTα gene and primer-R for 3′end of HA sequence; 3. Primer-F for initiation codon of CCTα gene and primer-R for a sequence of BTU1 gene where the fragment was intended to recombine. B) PCR analysis of full coding sequence (using AlfF and AlfR primers that anneal respectively at initiation and termination codons) of CCTα showing the presence of cDNA CCTα (1.6-kb) and a CCTα fragment with size ∼3.5-kb corresponding to the neo-disrupted-CCT allele present in the native locus of the rescued CCTα-HA strain. A heterozygous strain (HZ), containing the genomic wildtype (WT) CCTα allele (2.8-kb) and the disrupted allele (3.5-kb), was used to compare PCR band pattern. WT strain and plasmid DNA containing the cDNA of CCTα (C+) were also used as controls. C) PCR analysis of the macronuclear genotype of transformed CCTα-mutG346E strain. PCR products obtained using AlfF and AlfR primers that anneal respectively at initiation and termination codons in WT cells, HZ cells (that have in their macronuclear genotype the wildtype and neo-disrupted CCTα alleles) and the CCTα-mutG346E strain.
(0.09 MB TIF)

Figure S5 CCTα is a ciliary protein found in both axonemal and membrane/matrix fraction of cilia. A) Cilia from wildtype cells were isolated and fractionated in axonemal (Ax) and membranar (Mb) fraction which contains the soluble ciliary matrix. Western blot analysis using a serum against CCTα was performed showing the presence of the protein in both ciliary fractions and in total cilia extract. Western blot using anti α-tubulin supports the effectiveness of the cilia fractionation. B) The specificity of the antibody used above was confirmed by preabsorption of the antibody with the peptide used to elicit it. Western blot analysis of total protein extracts of wildtype cells and purified cilia extracts revealed only one specific band for CCTα that is not detected when antibody is pre-absorb to the peptide.
(0.19 MB TIF)

Figure S6 The apical domain of CCTα is related to a domain in BBS6 protein and contains a highly conserved G346 residue. A) Multiple sequence alignment of BBS6 and CCTα protein sequences using T-Coffee method. The multiple sequence alignment was produced with ClustalW2 program. The sequences were obtained from NCBI databases (see table S2). The alignment was edited with GeneDoc program and the aminoacid conserved percentage is indicated using the following shade style identity: red 100%; green 80% blue 60%. The position of the mutated G346 amino acid in this study is indicated by a black arrow. B) Schematic representation of CCTα protein showing its different domains, along with the position of the mutation made in the protein. C) Rasmol representation of the secondary structure of CCTα apical domain in wildtype and mutant cells (mutation G346E) using a ribbon model. The aminoacid residue that was mutagenized is depicted as white space-filling form (indicated with a red arrowhead). Note the mutation has led to the disappearance of β-sheets present in the ribbon model of wildtype cells (white arrow).
(3.12 MB TIF)

Materials and Methods S1 Contains Supplementary Materials and Methods S1; Deciliation of CCT depleted cells in a small scale.
(0.03 MB DOC)

Materials and Methods S2 Contains Supplementary Material and Methods S2; Cilia isolation and fractionation.
(0.03 MB DOC)

References S1 Contains the References S1 of supplementary data.
(0.03 MB DOC)

References S2 Contains the References S2 of supplementary data.
(0.03 MB DOC)

Table S1 Supplementary data.
(0.05 MB DOC)

Table S2 Supplementary data.
(0.04 MB DOC)

## References

[1] Kozminski KG, Johnson KA, Forscher P, Rosenbaum JL (1993). A motility in the eukaryotic flagellum unrelated to flagellar beating.. Proceedings of the National Academy of Sciences of the United States of America.

[2] Kozminski K, Beech P, Rosenbaum J (1995). The Chlamydomonas kinesin-like protein FLA10 is involved in motility associated with the flagellar membrane.. J Cell Biol.

[3] Cole DG, Diener DR, Himelblau AL, Beech PL, Fuster JC (1998). Chlamydomonas Kinesin-II-dependent Intraflagellar Transport (IFT): IFT Particles Contain Proteins Required for Ciliary Assembly in Caenorhabditis elegans Sensory Neurons.. J Cell Biol.

[4] Pazour GJ, Dickert BL, Witman GB (1999). The DHC1b (DHC2) Isoform of Cytoplasmic Dynein Is Required for Flagellar Assembly.. J Cell Biol.

[5] Porter ME, Bower R, Knott JA, Byrd P, Dentler W (1999). Cytoplasmic Dynein Heavy Chain 1b Is Required for Flagellar Assembly in Chlamydomonas.. Mol Biol Cell.

[6] Yang C, Compton MM, Yang P (2005). Dimeric Novel HSP40 Is Incorporated into the Radial Spoke Complex during the Assembly Process in Flagella.. Mol Biol Cell.

[7] Bloch M, Johnson K (1995). Identification of a molecular chaperone in the eukaryotic flagellum and its localization to the site of microtubule assembly.. J Cell Sci.

[8] Casano C, Gianguzza F, Roccheri MC, Di Giorgi R, Maenza L (2003). Hsp40 Is Involved in Cilia Regeneration in Sea Urchin Embryos.. J Histochem Cytochem.

[9] Stephens RE, Lemieux NA (1999). Molecular chaperones in cilia and flagella: Implications for protein turnover.. Cell Motility and the Cytoskeleton.

[10] Williams N, Nelsen E (1997). HSP70 and HSP90 homologs are associated with tubulin in hetero-oligomeric complexes, cilia and the cortex of Tetrahymena.. J Cell Sci.

[11] Satouh Y, Padma P, Toda T, Satoh N, Ide H (2005). Molecular Characterization of Radial Spoke Subcomplex Containing Radial Spoke Protein 3 and Heat Shock Protein 40 in Sperm Flagella of the Ascidian Ciona intestinalis.. Mol Biol Cell.

[12] Yang C, Owen HA, Yang P (2008). Dimeric heat shock protein 40 binds radial spokes for generating coupled power strokes and recovery strokes of 9+2 flagella.. J Cell Biol.

[13] Kim JC, Ou YY, Badano JL, Esmail MA, Leitch CC (2005). MKKS/BBS6, a divergent chaperonin-like protein linked to the obesity disorder Bardet-Biedl syndrome, is a novel centrosomal component required for cytokinesis.. J Cell Sci.

[14] Soares H, Penque D, Mouta C, Rodrigues-Pousada C (1994). A Tetrahymena orthologue of the mouse chaperonin subunit CCT gamma and its coexpression with tubulin during cilia recovery.. Journal of Biological Chemistry.

[15] Cyrne L, Guerreiro P, Cardoso AC, Rodrigues-Pousada C, Soares H (1996). The Tetrahymena chaperonin subunit CCT[eta] gene is coexpressed with CCT[gamma] gene during cilia biogenesis and cell sexual reproduction.. FEBS Letters.

[16] Seixas C, Casalou C, Melo LV, Nolasco S, Brogueira P (2003). Subunits of the chaperonin CCT are associated with Tetrahymena microtubule structures and are involved in cilia biogenesis.. Experimental Cell Research.

[17] Dunn AY, Melville MW, Frydman J (2001). Review: Cellular Substrates of the Eukaryotic Chaperonin TRiC/CCT.. Journal of Structural Biology.

[18] Pappenberger G, Wilsher JA, Mark Roe S, Counsell DJ, Willison KR (2002). Crystal Structure of the CCT[gamma] Apical Domain: Implications for Substrate Binding to the Eukaryotic Cytosolic Chaperonin.. Journal of Molecular Biology.

[19] Frydman J NE, Erdjument-Bromage H, Wall JS, Tempst P, Hartl FU (1992). Function in protein folding of TRiC, a cytosolic ring complex containing TCP-1and structurally related subunits.. EMBO J.

[20] Yaffe MB, Farr GW, Miklos D, Horwich AL, Sternlicht ML (1992). TCP1 complex is a molecular chaperone in tubulin biogenesis.. Nature.

[21] Melki R, Vainberg I, Chow R, Cowan N (1993). Chaperonin-mediated folding of vertebrate actin-related protein and gamma-tubulin.. J Cell Biol.

[22] Gao Y, Thomas JO, Chow RL, Lee G-H, Cowan NJ (1992). A cytoplasmic chaperonin that catalyzes [beta]-actin folding.. Cell.

[23] Frydman J, Hartl U (1996). Principles of Chaperone-Assisted Protein Folding: Differences Between in Vitro and in Vivo Mechanisms. [Report].. Science June.

[24] Gaertig J, Gu L, Hai B, Gorovsky MA (1994). High frequency vector-mediated transformation and gene replacement in Tetrahymena.. Nucl Acids Res.

[25] Orias E, Rasmussen L (1976). Dual capacity for nutrient uptake in Tetrahymena: IV. Growth without food vacuoles and its implications.. Experimental Cell Research.

[26] Brown JM, Marsala C, Kosoy R, Gaertig J (1999). Kinesin-II Is Preferentially Targeted to Assembling Cilia and Is Required for Ciliogenesis and Normal Cytokinesis in Tetrahymena.. Mol Biol Cell.

[27] Brown JM, Fine NA, Pandiyan G, Thazhath R, Gaertig J (2003). Hypoxia Regulates Assembly of Cilia in Suppressors of Tetrahymena Lacking an Intraflagellar Transport Subunit Gene.. Mol Biol Cell.

[28] Shang Y, Song X, Bowen J, Corstanje R, Gao Y (2002). A robust inducible-repressible promoter greatly facilitates gene knockouts, conditional expression, and overexpression of homologous and heterologous genes in Tetrahymenathermophila.. Proceedings of the National Academy of Sciences of the United States of America.

[29] Soares H CL, Casalou C, Ehmann B, Rodrigues-Pousada C (1997). The third member of the Tetrahymena CCT subunit gene family, TpCCT alpha, encodes a component of the hetero-oligomeric chaperonin complex.. Biochem J.

[30] Gómez-Puertas P, Martín-Benito J, Carrascosa JL, Willison KR, Valpuesta JM (2004). The substrate recognition mechanisms in chaperonins.. Journal of Molecular Recognition.

[31] Schwede T, Kopp J, Guex N, Peitsch MC (2003). SWISS-MODEL: an automated protein homology-modeling server.. Nucl Acids Res.

[32] Ditzel L, Löwe J, Stock D, Stetter K-O, Huber H (1998). Crystal Structure of the Thermosome, the Archaeal Chaperonin and Homolog of CCT..

[33] Ursic D, Culbertson MR (1991). The yeast homolog to mouse Tcp-1 affects microtubule-mediated processes.. Mol Cell Biol.

[34] Vinh DB, Drubin DG (1994). A yeast TCP-1-like protein is required for actin function in vivo.. Proceedings of the National Academy of Sciences of the United States of America.

[35] Dekker C, Stirling PC, McCormack EA, Filmore H, Paul A (2008). The interaction network of the chaperonin CCT.. EMBO J.

[36] Gong Y, Kakihara Y, Krogan N, Greenblatt J, Emili A (2009). An atlas of chaperone-protein interactions in Saccharomyces cerevisiae: implications to protein folding pathways in the cell.. Mol Syst Biol.

[37] Grantham J, Brackley KI, Willison KR (2006). Substantial CCT activity is required for cell cycle progression and cytoskeletal organization in mammalian cells.. Experimental Cell Research.

[38] Wang H-W, Nogales E (2005). Nucleotide-dependent bending flexibility of tubulin regulates microtubule assembly.. Nature.

[39] Stolc V, Samanta MP, Tongprasit W, Marshall WF (2005). Genome-wide transcriptional analysis of flagellar regeneration in Chlamydomonas reinhardtii identifies orthologs of ciliary disease genes.. Proceedings of the National Academy of Sciences of the United States of America.

[40] Keller LC, Romijn EP, Zamora I, Yates JR, Marshall WF (2005). Proteomic Analysis of Isolated Chlamydomonas Centrioles Reveals Orthologs of Ciliary-Disease Genes.. Current Biology.

[41] Stephens RE (1997). Synthesis and Turnover of Embryonic Sea Urchin Ciliary Proteins during Selective Inhibition of Tubulin Synthesis and Assembly.. Mol Biol Cell.

[42] Piperno G, Mead K (1997). Transport of a novel complex in the cytoplasmic matrix of Chlamydomonas flagella.. Proceedings of the National Academy of Sciences of the United States of America.

[43] Mitchell BF, Pedersen LB, Feely M, Rosenbaum JL, Mitchell DR (2005). ATP Production in Chlamydomonas reinhardtii Flagella by Glycolytic Enzymes.. Mol Biol Cell.

[44] Kato-Minoura T, Hirono M, Kamiya R (1997). Chlamydomonas Inner-Arm Dynein Mutant, ida5, Has a Mutation in an Actin-encoding Gene.. J Cell Biol.

[45] Williams NE, Tsao C-C, Bowen J, Hehman GL, Williams RJ (2006). The Actin Gene ACT1 Is Required for Phagocytosis, Motility, and Cell Separation of Tetrahymena thermophila.. Eukaryotic Cell.

[46] Kim SK, Park TJ, Abitua PB, Wallingford JB (2009). The PCP effector Fritz governs microtubule assembly and ciliogenesis in vertebrate multi-ciliated cells.. Developmental Biology.

[47] Skriver L, Williams N (1980). Regeneration of cilia in starved Tetrahymena thermophila involves induced synthesis of ciliary proteins but not synthesis of membrane lipids.. Biochem J.

[48] Dentler W, Rosenbaum J (1977). Flagellar elongation and shortening in Chlamydomonas. III. structures attached to the tips of flagellar microtubules and their relationship to the directionality of flagellar microtubule assembly.. J Cell Biol.

[49] Dentler W, LeCluyse E (1982). Microtubule capping structures at the tips of tracheal cilia: evidence for their firm attachment during ciliary bend formation and the restriction of microtubule sliding.. Cell Motility and the Cytoskeleton.

[50] Dentler W (1984). Attachment of the cap to the central microtubules of Tetrahymena cilia.. J Cell Sci.

[51] Portman R, LeCluyse E, Dentler W (1987). Development of microtubule capping structures in ciliated epithelial cells.. J Cell Sci.

[52] Miller J, Wang W, Balczon R, Dentler W (1990). Ciliary microtubule capping structures contain a mammalian kinetochore antigen.. J Cell Biol.

[53] Roobol A, Sahyoun ZP, Carden MJ (1999). Selected Subunits of the Cytosolic Chaperonin Associate with Microtubules Assembled in Vitro.. Journal of Biological Chemistry.

[54] Grantham J, Ruddock LW, Roobol A, Carden MJ (2002). Eukaryotic chaperonin containing T-complex polypeptide 1 interacts with filamentous actin and reduces the initial rate of actin polymerization in vitro.. Cell Stress & Chaperones.

[55] Johnson K, Rosenbaum J (1992). Polarity of flagellar assembly in Chlamydomonas.. J Cell Biol.

[56] Creutz CE, Liou A, Snyder SL, Brownawell A, Willison K (1994). Identification of the major chromaffin granule-binding protein, chromobindin A, as the cytosolic chaperonin CCT (chaperonin containing TCP-1).. Journal of Biological Chemistry.

[57] Wagner CT, Lu IY, Hoffman MH, Sun WQ, Trent JD (2004). T-complex Polypeptide-1 Interacts with the Erythrocyte Cytoskeleton in Response to Elevated Temperatures.. Journal of Biological Chemistry.

[58] Nachury MV, Loktev AV, Zhang Q, Westlake CJ, Peränen J (2007). A Core Complex of BBS Proteins Cooperates with the GTPase Rab8 to Promote Ciliary Membrane Biogenesis.. Cell.

[59] Seo S, Baye LM, Schulz NP, Beck JS, Zhang Q (2010). BBS6, BBS10, and BBS12 form a complex with CCT/TRiC family chaperonins and mediate BBSome assembly.. Proceedings of the National Academy of Sciences.

[60] Shah AS, Farmen SL, Moninger TO, Businga TR, Andrews MP (2008). Loss of Bardet–Biedl syndrome proteins alters the morphology and function of motile cilia in airway epithelia.. Proceedings of the National Academy of Sciences.

[61] Gorovsky M (1973). Macro- and micronuclei of Tetrahymena pyriformis: a model system for studying the structure and function of eukaryotic nuclei.. J Protozool.

[62] Cassidy-Hanley D, Bowen J, Lee JH, Cole E, VerPlank LA (1997). Germline and Somatic Transformation of Mating Tetrahymena thermophila by Particle Bombardment.. Genetics.

[63] Hai B, Gorovsky MA (1997). Germ-line knockout heterokaryons of an essential α-tubulin gene enable high-frequency gene replacement and a test of gene transfer from somatic to germ-line nuclei in Tetrahymena thermophila.. Proceedings of the National Academy of Sciences of the United States of America.

[64] Kunkel TA (1985). Rapid and efficient site-specific mutagenesis without phenotypic selection.. Proceedings of the National Academy of Sciences of the United States of America.

[65] Bruns P, Cassidy-Hanley D, Asai D, Forney J (2000). Biolistic transformation of macro- and micronuclei..

[66] Hai B, Gaertig J, Gorovsky M, Asai D, Forney J (2000). Knockout heterokaryons enable facile mutagenic analysis of essential genes in Tetrahymena..

[67] Thazhath R, Liu C, Gaertig J (2002). Polyglycylation domain of [beta]-tubulin maintains axonemal architecture and affects cytokinesis in Tetrahymena.. Nat Cell Biol.

[68] Notredame C, Higgins DG, Heringa J (2000). T-coffee: a novel method for fast and accurate multiple sequence alignment.. Journal of Molecular Biology.

