# Supplementary figures and images for: CCTα and CCTδ Chaperonin Subunits Are Essential and Required for Cilia Assembly and Maintenance in Tetrahymena
Source: PLoS One. 2010 May 18;5(5):e10704. doi: 10.1371/journal.pone.0010704 (PMC2872681; doi:10.1371/journal.pone.0010704)

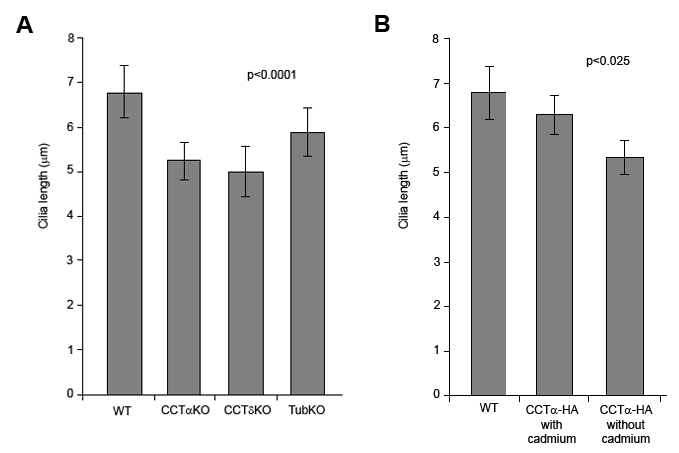

Supplement: Figure S1 — Cilia length in cells with low levels of CCTα and CCTδ. A) Cilia length in a population of wildtype, CCTα and δ KOs, or tubulin depleted cells were measured after 26 hpm. Cilia are significantly shorter in the absence of the referred CCT subunits, with p<0.0001. B) Cilia length in a population of wild type cells or in a population of rescued CCTα-HA cells, grown in medium with or without cadmium, was measured. Cilia are significantly shorter in rescued CCTα-HA cells grown without cadmium, with p<0,025, which correlates with the absence of CCTα in cilia (see Fig. 2H). Number of measured cilia was 145 in wildtype cells, n = 196 in CCTδ-KO cells, n = 35 in CCTα-KO (with similar values at 36 hpm with n = 112), n = 102 in tubulin KO cells, n = 90 in CCTα-HA cells growing with cadmium and n = 83 cilia in CCTα-HA cells growing without cadmium. (0.07 MB TIF) [file pone.0010704.s001.tif]

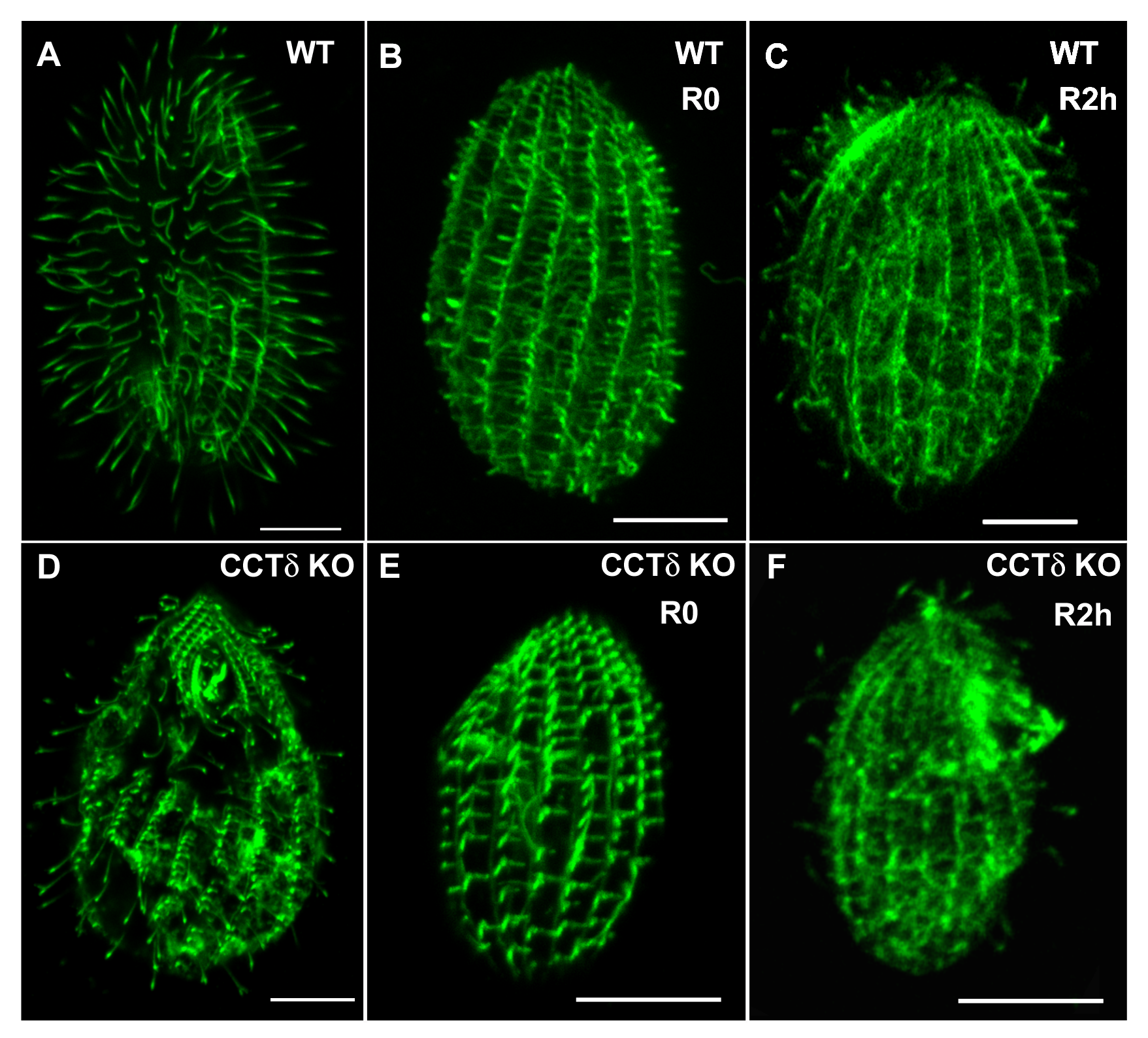

Supplement: Figure S2 — CCTδ depleted cells are unable to reciliate. A) Confocal immunofluorescence of αtubulin (using 12G10 monoclonal antibody) in Tetrahymena wildtype (WT) and CCTδ KO cells to analyze their reciliation capacity. (A and D) Non-deciliated cells (WT) and CCTδ KO cell. (B and E) Cells analyzed immediately after deciliation (R0); (C and F) Cells analyzed after 2 h of reciliation (R2h). WT cells are able to full reciliate and recover their swimming capacity (not shown), while CCT depleted cells are mostly unable to reciliate their cilia, or randomly recover a few cilia. Note the apparent gaps in transversal microtubules present in the CCT-KO cell. Scale bar = 10 µm. (1.87 MB TIF) [file pone.0010704.s002.tif]

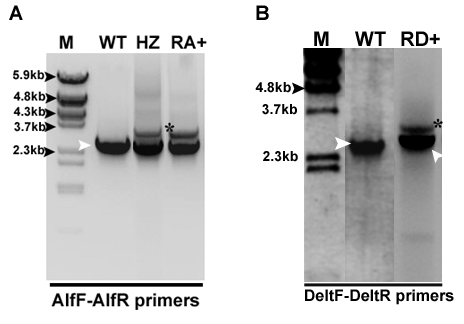

Supplement: Figure S3 — PCR analysis of strains obtained in rescue experiments to confirm their genotype. A) Analysis by PCR of the CCTα locus in wildtype cells and rescued CCTα-KO cells. For WT strain it was observed only one band (white arrowhead) corresponding to WT allele, whereas in rescued CCTα KO strain (RA+) an additional band (asterisk) corresponding to the disrupted-allele CCTα is visible. To facilitate the interpretation of the bands pattern a heterozygous strain for CCTα disruption was obtained by a cross of one of the CCTα-neo-disrupted heterokaryon strains with a WT strain. In the heterozygous (HZ) two bands were found, one corresponding to the WT allele (2.8 kb) and the other corresponding to the disrupted-allele of CCTα, with the expected size (3.5 kb). B) Analysis by PCR of the CCTδ locus in WT cells and in the rescued CCTδ-KO cells. Also, PCR analysis revealed two bands in rescued CCTδ-KO strain (RD+) confirming the presence of the WT and the disrupted allele. (0.09 MB TIF) [file pone.0010704.s003.tif]

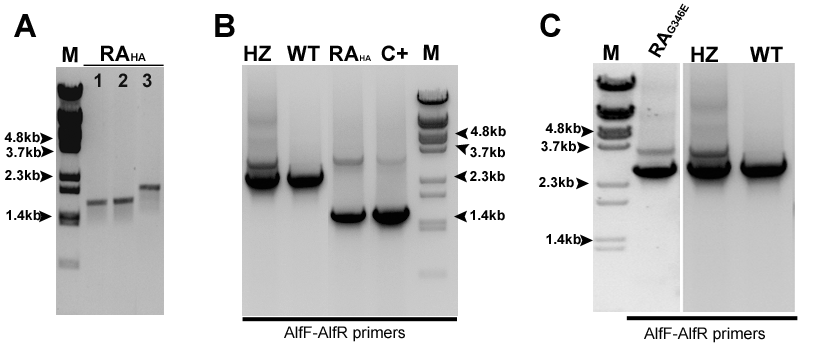

Supplement: Figure S4 — Genotypic analysis of the CCTα-KO cells rescued with a HA tagged CCTα cDNA or genomic CCTαmutG346E. A) PCR analysis using genomic DNA from the rescued CCTα-HA strain (RAHA) with the: 1. pair of primers that amplify full cDNA CCTα; 2. Primer-F for initiation codon of CCTα gene and primer-R for 3′end of HA sequence; 3. Primer-F for initiation codon of CCTα gene and primer-R for a sequence of BTU1 gene where the fragment was intended to recombine. B) PCR analysis of full coding sequence (using AlfF and AlfR primers that anneal respectively at initiation and termination codons) of CCTα showing the presence of cDNA CCTα (1.6-kb) and a CCTα fragment with size ∼3.5-kb corresponding to the neo-disrupted-CCT allele present in the native locus of the rescued CCTα-HA strain. A heterozygous strain (HZ), containing the genomic wildtype (WT) CCTα allele (2.8-kb) and the disrupted allele (3.5-kb), was used to compare PCR band pattern. WT strain and plasmid DNA containing the cDNA of CCTα (C+) were also used as controls. C) PCR analysis of the macronuclear genotype of transformed CCTα-mutG346E strain. PCR products obtained using AlfF and AlfR primers that anneal respectively at initiation and termination codons in WT cells, HZ cells (that have in their macronuclear genotype the wildtype and neo-disrupted CCTα alleles) and the CCTα-mutG346E strain. (0.09 MB TIF) [file pone.0010704.s004.tif]

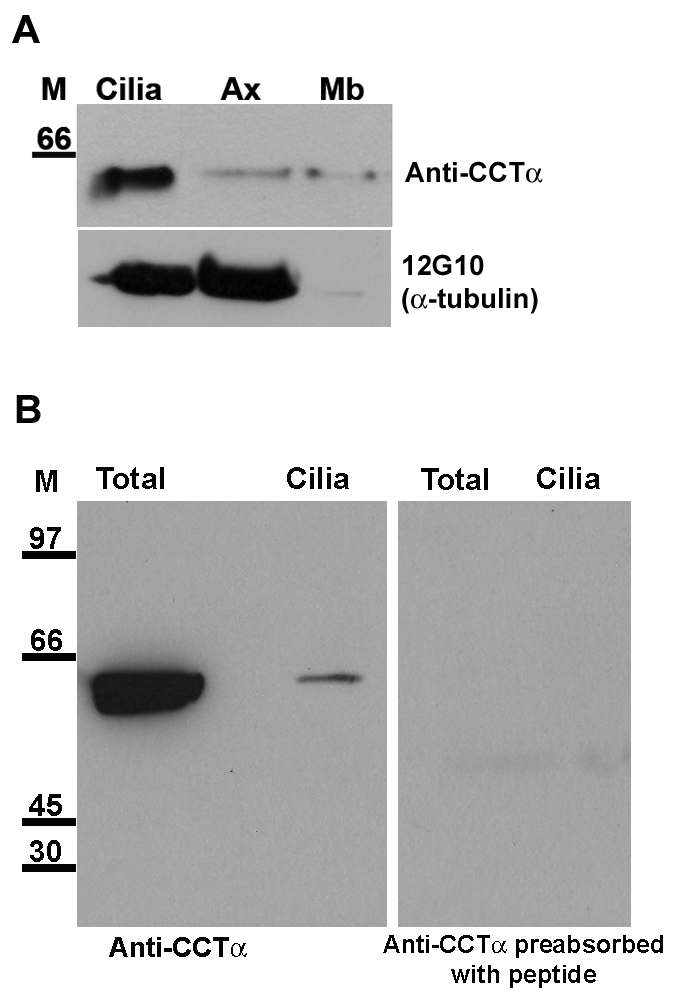

Supplement: Figure S5 — CCTα is a ciliary protein found in both axonemal and membrane/matrix fraction of cilia. A) Cilia from wildtype cells were isolated and fractionated in axonemal (Ax) and membranar (Mb) fraction which contains the soluble ciliary matrix. Western blot analysis using a serum against CCTα was performed showing the presence of the protein in both ciliary fractions and in total cilia extract. Western blot using anti α-tubulin supports the effectiveness of the cilia fractionation. B) The specificity of the antibody used above was confirmed by preabsorption of the antibody with the peptide used to elicit it. Western blot analysis of total protein extracts of wildtype cells and purified cilia extracts revealed only one specific band for CCTα that is not detected when antibody is pre-absorb to the peptide. (0.19 MB TIF) [file pone.0010704.s005.tif]

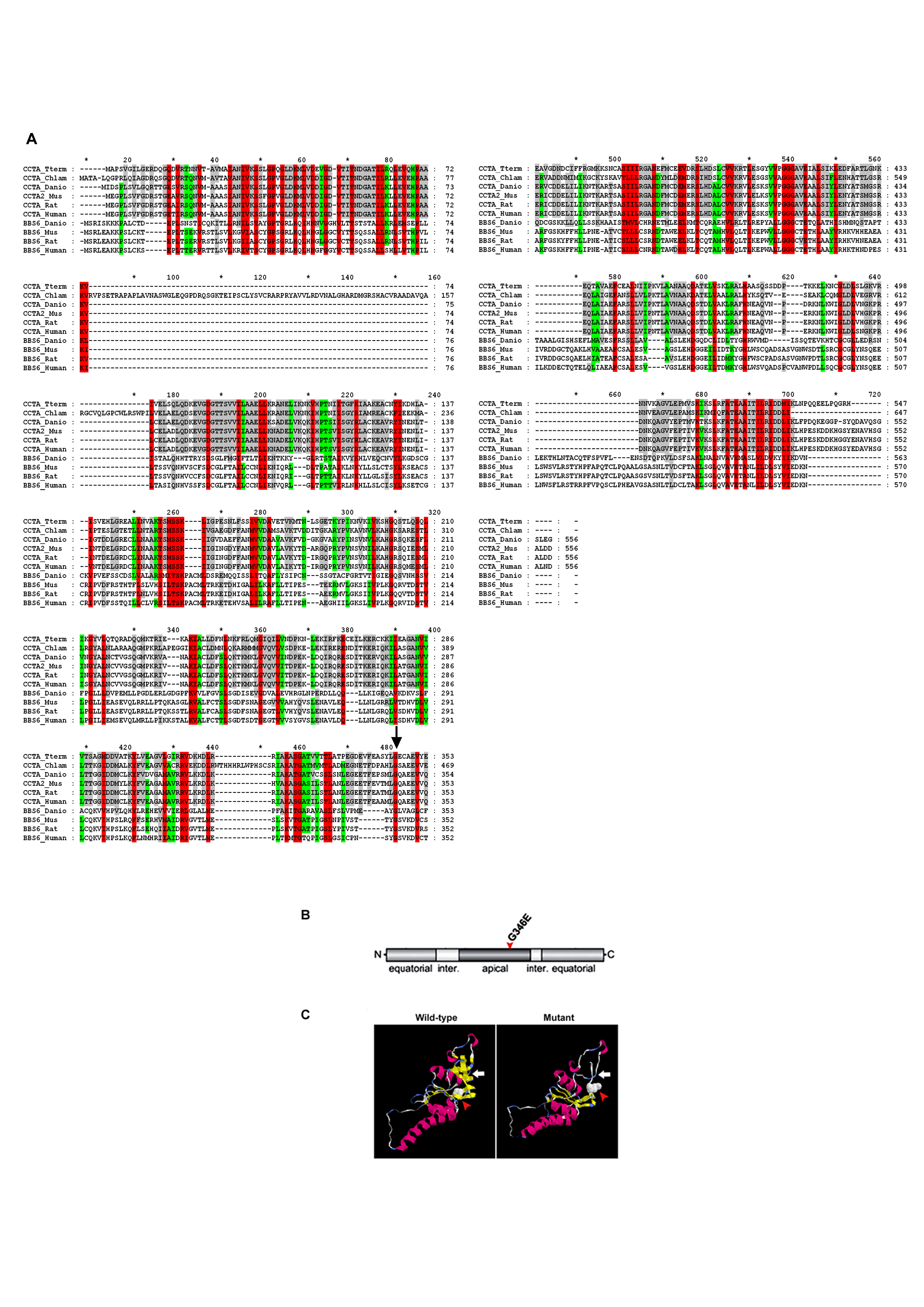

Supplement: Figure S6 — The apical domain of CCTα is related to a domain in BBS6 protein and contains a highly conserved G346 residue. A) Multiple sequence alignment of BBS6 and CCTα protein sequences using T-Coffee method. The multiple sequence alignment was produced with ClustalW2 program. The sequences were obtained from NCBI databases (see table S2). The alignment was edited with GeneDoc program and the aminoacid conserved percentage is indicated using the following shade style identity: red 100%; green 80% blue 60%. The position of the mutated G346 amino acid in this study is indicated by a black arrow. B) Schematic representation of CCTα protein showing its different domains, along with the position of the mutation made in the protein. C) Rasmol representation of the secondary structure of CCTα apical domain in wildtype and mutant cells (mutation G346E) using a ribbon model. The aminoacid residue that was mutagenized is depicted as white space-filling form (indicated with a red arrowhead). Note the mutation has led to the disappearance of β-sheets present in the ribbon model of wildtype cells (white arrow). (3.12 MB TIF) [file pone.0010704.s006.tif]
