# Supplementary material for: CCTα and CCTδ Chaperonin Subunits Are Essential and Required for Cilia Assembly and Maintenance in Tetrahymena
Source: PLoS One. 2010 May 18;5(5):e10704. doi: 10.1371/journal.pone.0010704 (PMC2872681; doi:10.1371/journal.pone.0010704)
Supplement: Materials and Methods S1 — Contains Supplementary Materials and Methods S1; Deciliation of CCT depleted cells in a small scale. (0.03 MB DOC) [file pone.0010704.s007.doc]

**MATERIALS AND METHODS S1**

*Deciliation of CCT depleted cells in a small scale*

To allow deciliation of a small number of KO-cells, we adapted the protocol described by Calzone and Gorovsky [References S1]. Approximately 20 cells were isolated from drop cultures and placed into a 30 µl size drop of deciliation solution (10 % Ficoll 400, 10 mM sodium acetate (CH_3_COONa,), 10 mM CaCl_2_, 10 mM EDTA pH 4.2) for 1 min, while mixing by a suction-based needle. Cells were collected and transferred into a drop containing 10 mM Tris-HCl pH 7.5 and allowed to reciliate at 30ºC in a moist chamber.
