# Supplementary material for: CCTα and CCTδ Chaperonin Subunits Are Essential and Required for Cilia Assembly and Maintenance in Tetrahymena
Source: PLoS One. 2010 May 18;5(5):e10704. doi: 10.1371/journal.pone.0010704 (PMC2872681; doi:10.1371/journal.pone.0010704)
Supplement: Materials and Methods S2 — Contains Supplementary Material and Methods S2; Cilia isolation and fractionation. (0.03 MB DOC) [file pone.0010704.s008.doc]

**Materials and Methods S2**

*Cilia isolation and fractionation*

Cilia were isolated from *Tetrahymena* cultures (2x10^5^ cells/ml of density) by the dibucaine method [References S2]. Briefly, cells were harvested by centrifugation at room temperature at 1500 xg for 3 min, washed twice and ressuspended in HNMK solution (50 mM Hepes-KOH, pH 6.9; 36 mM NaCl; 0.1 mM MgSO_4_; 1 mM KCl). Cells were concentrated 10-fold in HNMKS (HNMK complemented with 8.5% sucrose). After brief swirling of the cells, dibucaine and CaCl_2_ were added to final concentration of 3 mM and 10 mM, respectively. Gently, cells were moved up and down through a thin glass pipette during 3 min to allow homogenization and deciliation. Cells were then diluted in HNMK solution to their initial volume. The deciliated cell bodies were pelleted by centrifugation at 1500 xg for 5 min. Supernatant was cleaned from eventual cell bodies by two successive centrifugation at 2000xg for 5 min at 4ºC (all subsequent steps were performed at 4°C). Cilia present in the supernatant were pelleted at 10 0000 g during 30 min. The pellet of cilia was ressuspended in 1 ml of buffer B (50 mM Hepes-KOH, pH 7.4; 1 mM EDTA; 1 mM EGTA; 3 mM MgCl_2_; 250 mM sucrose; 0.1 mM DTT) and centrifuged 4 min at 600xg to remove possible cell debris. This last step was repeated usually twice more until the preparation was judged to be >99% cilia, as observed by phase microscopy. The fractionation of cilia was performed as described below [52]. Cilia were pelleted at 10 0000 g during 30 min and ressuspended in TMESDP (25 mM Tris-HCI, pH 7.4, 3 mM MgSO_4_, 0.1 mM EGTA, 250 mM sucrose, 1 mM DTT, 0.3 mM PMSF). Cilia were demembranated by suspending them in TMESDP complemented with 0.5% Triton X-100 and incubating the suspension for 20 min at 4ºC. Axonemes were sedimented at 17,400 g at 4ºC and then resuspended in TMESDP.
