# Supplementary material for: CCTα and CCTδ Chaperonin Subunits Are Essential and Required for Cilia Assembly and Maintenance in Tetrahymena
Source: PLoS One. 2010 May 18;5(5):e10704. doi: 10.1371/journal.pone.0010704 (PMC2872681; doi:10.1371/journal.pone.0010704)
Supplement: References S1 — Contains the References S1 of supplementary data. (0.03 MB DOC) [file pone.0010704.s009.doc]

**REFERENCES S1.**

S1-Calzone F, Gorovsky M (1982) Cilia regeneration in Tetrahymena. A simple reproducible method for producing large numbers of regenerating cells. Exp Cell Res 140: 471-476.
