# Supplementary material for: CCTα and CCTδ Chaperonin Subunits Are Essential and Required for Cilia Assembly and Maintenance in Tetrahymena
Source: PLoS One. 2010 May 18;5(5):e10704. doi: 10.1371/journal.pone.0010704 (PMC2872681; doi:10.1371/journal.pone.0010704)
Supplement: References S2 — Contains the References S2 of supplementary data. (0.03 MB DOC) [file pone.0010704.s010.doc]

**REFERENCES S2**

S2. Thompson GA, Baugh LC, Walker LF (1974) Nonlethal Deciliation of Tetrahymena by a local anesthetic and its utility as a tool for studying cilia regeneration. The Journal of Cell Biology 61: 253-257.
