# Supplementary material for: CCTα and CCTδ Chaperonin Subunits Are Essential and Required for Cilia Assembly and Maintenance in Tetrahymena
Source: PLoS One. 2010 May 18;5(5):e10704. doi: 10.1371/journal.pone.0010704 (PMC2872681; doi:10.1371/journal.pone.0010704)
Supplement: Table S1 — Supplementary data. (0.05 MB DOC) [file pone.0010704.s011.doc]

**Table S1. Strains used in this study, their genotype and phenotype characteristics**

| Strain | Micronuclear Genotype | Macronuclear Genotype | Macronuclear Phenotype: drug resistances and mating types |
| --- | --- | --- | --- |
| B2086.1 | WT | WT | Mat II |
| CU428.1 | *mpr1-1/mpr1-1* | WT | mp-s; mat VII |
| CU427.3 | *chx1-1/chx1-1* | WT | cy-s; mat VI |
| CU522 | *btu1-K350M/btu1-K350M* | *btu1-K350M* | taxol-s; mat n/d |
| B*VII | Non-functional mic (star) | WT | Mat VII |
| A*III | Non-functional mic (star) | WT | Mat III |
| CCTD-1A | *CCTδ::neo2/CCTδ::neo2* | WT | pm-s; mat VII |
| CCTD-1B | *CCTδ::neo2/CCTδ::neo2* | WT | pm-s; mat IV |
| CCTD.1AA*-2 | *CCTδ::neo2/CCTδ::neo2* | WT | pm-s; mat VII |
| CCTD.1AA*-4A | *CCTδ::neo2/CCTδ::neo2* | WT | pm-s; mat III |
| CCTA-3A | CCTα::neo2/CCTα::neo2 | WT | pm-s; mat IV |
| CCTA-10A | CCTα::neo2/CCTα::neo2 | WT | pm-s; mat VII |
| CCTA-A1 | *CCTα::neo2/CCTα::neo2*; *mpr1-1/mpr1-1* | WT | pm-s; mp-s; mat IV |
| CCTA-B5 | *CCTα::neo2/CCTα::neo2; mpr1-1/mpr1-1* | WT | pm-s; mp-s; mat VII |
| CCTA.B5A*-3 | *CCTα::neo2/CCTα::neo2*  *mpr1-1/mpr1-1* | WT | pm-s; mp-s; mat VII |
| CCTA.B5A*-3A | *CCTα::neo2/CCTα::neo2;*  *mpr1-1/mpr1-1* | WT | pm-s; mp-s; mat III |

WT: wildtype; *chx1-1*:cycloheximide (cy)-resistant marker, *btu1-K350M:* mutation of *BTU1* gene that confers hypersensitivity at the microtubule-stabilizing drug taxol*; mpr1-1*: 6-methylpurine (mp)-resistant marker; mp-s: 6-methylpurine sensitive; cy-s: cycloheximide sensitive; pm-s: paromomycin sensitive; taxol-s: taxol sensitive; mat designates the mating type of the strain; n/d- not determined.
