# Supplementary material for: CCTα and CCTδ Chaperonin Subunits Are Essential and Required for Cilia Assembly and Maintenance in Tetrahymena
Source: PLoS One. 2010 May 18;5(5):e10704. doi: 10.1371/journal.pone.0010704 (PMC2872681; doi:10.1371/journal.pone.0010704)
Supplement: Table S2 — Supplementary data. (0.04 MB DOC) [file pone.0010704.s012.doc]

**Table S2. Pubmed accession numbers of CCTα** **and BBS6 protein sequences used in a multiple sequence alignment.**

| Abbreviation used in the alignment | Specie name | Accession Number |
| --- | --- | --- |
| CCTA_Ttherm | *Tetrahymena thermophila* | EAR90580 |
| CCTA_Chlam | *Chlamydomonas reinhardtii* | EDP05815 |
| CCTA_Danio | *Danio rerio* | Q9W792 |
| CCTA_Mus | *Mus musculus* | P11983 |
| CCTA_Rat | Rattus norvegicus | P28480 |
| CCTA_human | *Homo sapiens* | P17987 |
| BBS6_Danio | *Danio rerio* | AAH45401 |
| BBS6_Mus | *Mus mus* | AAH24359 |
| BBS6_Rat | Rattus norvegicus | NP_001008354 |
| BBS6_Human | *Homo sapiens* | Q9NPJ1 |
